# Endogenous PTEN acts as the key determinant for mTOR inhibitor sensitivity by inducing the stress-sensitized PTEN-mediated death axis in KSHV-associated malignant cells

**DOI:** 10.3389/fmolb.2023.1062462

**Published:** 2023-08-02

**Authors:** Piyanki Das, Sonali Pal, Nilanjana Das, Koushik Chakraborty, Koustav Chatterjee, Sudipa Mal, Tathagata Choudhuri

**Affiliations:** Department of Biotechnology, Visva-Bharati, Santiniketan, West Bengal, India

**Keywords:** PTEN, mTOR inhibitors, drug sensitivity determinant, KSHV-associated malignancy, stress, PTEN-mediated cell death

## Abstract

As a part of viral cancer evolution, KSHV-infected human endothelial cells exert a unique transcriptional program *via* upregulated mTORC1 signaling. This event makes them sensitive to mTOR inhibitors. Master transcriptional regulator PTEN acts as the prime regulator of mTOR and determining factor for mTOR inhibitory drug resistance and sensitivity. PTEN is post-translationally modified in KSHV-associated cell lines and infected tissues. Our current study is an attempt to understand the functional role of upstream modulator PTEN in determining the sensitivity of mTOR inhibitors against KSHV-infected cells in an *in vitro* stress-responsive model. Our analysis shows that, despite phosphorylation, endogenous levels of intact PTEN in different KSHV-infected cells compared to normal and non-infected cells are quite high. Genetic overexpression of intact PTEN showed functional integrity of this gene in the infected cells in terms of induction of a synchronized cell death process *via* cell cycle regulation and mitochondria-mediated apoptosis. PTEN overexpression enhanced the mTOR inhibitory drug activity, the silencing of which hampers the process against KSHV-infected cells. Additionally, we have shown that endogenous PTEN acts as a stress balancer molecule inside KSHV-infected cells and can induce stress-sensitized death program post mTOR inhibitor treatment, lined up in the ATM-chk2-p53 axis. Moreover, autophagic regulation was found as a major regulator in mTOR inhibitor-induced PTEN-mediated death axis from our study. The current work critically intersected the PTEN-mediated stress balancing mechanism where autophagy has been utilized as a part of the KSHV stress management system and is specifically fitted and switched toward autophagy-mediated apoptosis directing toward a therapeutic perspective.

## Introduction

The master regulatory cellular protein, phosphatase and tensin homolog, PTEN, acts as a major tumor suppressor gene by regulating vital cellular functioning related to cell survival and growth. In terms of its regulatory role over cellular signaling, PI3K/mTOR signaling is the principal one. Thus, the endogenous PTEN gene status becomes a classical oncogenic cellular marker for determining cancer. Different types of PTEN mutations, such as total deletion and promoter methylation modulation of expression, have been reported to be associated with different types of infected and non-infected cancer types (Jones et al., 2013; Khalid et al., 2017). KSHV (HHV-8)-associated primary effusion lymphoma, a deadly form of cancer without any fruitful therapeutic strategies available, is one such cancer where PTEN has been reported to be post-translationally modified and hyperphosphorylated (Roy and Dittmer, 2011). Hence, we aimed to study the impact of such endogenously expressed post-translationally modulated PTEN and its functional importance in terms of its therapeutic possibilities as an upstream master regulator of the mTOR pathway in KSHV-associated cancer cells.

In search of the functional importance of PTEN during cancer, it is observed that PTEN act as the negative regulator of PI3K/Akt/mTOR signaling. Thus, PTEN mutation results in an overactivated mammalian target of rapamycin (mTOR) signaling. mTOR is a protein kinase pathway, downstream of the PTEN regulatory cascade, and it controls several interlinked vital cellular regulatory signaling during cancer (Porta et al., 2014). mTOR inhibitors are widely reported and clinically applied as a fruitful therapeutic option against such cases to control the deregulated pathway (Zou et al., 2020). Different generations of mTOR inhibitors are available with potential anti-cancerous effects and are under clinical use or under trial (Benjamin et al., 2011). Parallelly, several cases of therapy-refractory cancer types are emerging day by day, where the mTOR pathway plays a vital role either by rescuing drug resistance or by distorting drug sensitivity (Jiang and Liu, 2008). On this note, development of drug resistance against mTOR inhibitors is a real threat that needs attention (Formisano et al., 2020). Since altered mTOR signaling is a well-known molecular therapeutic target point, research on the development of effective mTOR inhibitors has so far been critically addressed through various generations of this class of drugs. First-generation mTOR inhibitors mainly targeting mTORC1 have been developed in terms of better efficacy and sensitivity to second-generation inhibitors with dual effect against mTORC1 and C2 target specificity, and last, mTOR kinase inhibitors are applied as third-generation mTOR inhibitors, particularly designed and developed targeting the previous generation’s mTOR inhibitor-mediated resistance mutations (Flemming, 2016; Rodrik-Outmezguine et al., 2016; La Manna et al., 2020; Feng et al., 2021). On this note, another effective approach is well reported with the application of direct AKT inhibitors, which are applied to counteract the upstream molecule AKT and applied in combination with mTOR inhibitors to enhance their activity as a combinatorial approach. If we critically analyze the developmental pattern of these different generations of mTOR inhibitors, it is observed that they are effective as mTOR-specific kinase inhibitors targeting specific kinase domains in these pathways (Fan et al., 2017). Now, this specific kinase domain modulation development of resistance and intervention of mTOR kinase inhibitors points toward the development of resistance and evidence of therapy refract tory cases, and evidence of parallel evolution is a major burden (Formisano et al., 2020). Such an event indicates the probability of the existence of certain continuous conditions inside the cancer cells that makes them exhibit this mTOR kinase ligand-specific modulation, resulting in these cells being the significant therapeutic target for different mTOR inhibitors. Thus, we need to focus on the route causative factor of mTOR inhibitory drug resistance or sensitivity, and for that, the regulation of such a group of drugs needs to be critically understood. In relation to this analysis, PTEN, the supremo of mTOR signaling, needs to be critically considered as the determining factor. There are very few reports available on PTEN modulation and mTOR drug sensitivity. PTEN deletion has been reported as an enhancing factor for mTOR inhibitor’s sensitivity as the cell proliferation event is totally dependent on its downstream mTOR modulation (Neshat et al., 2001). So, in our study, we tried to figure out whether the functional impact of the modulated version of PTEN, inside KSHV-infected cells, acts as a determinant for mTOR inhibitors. It has been previously reported that drug sensitivity/resistance is a part of the unique herpesviral transcriptional program where mTORC1 activity has been found as a causative agent for rapamycin sensitivity, exerting a major therapeutic consequence in a herpesviral oncogenic setup (Chang and Ganem, 2013). The continuous process of evolution of drug resistance makes an effective drug failure to execute persistence and complete cancer control. Following the rule of continuous and diverse evolution, a cancer cell can evolve itself in a tumor microenvironment. Genetic factors are important in this process. Such route of evolution is very common in viral pathogenic diseases. Herpesvirus, specifically KSHV/HHV-8, has been known to exert some unique transcriptional programs which make the cell sensitize against mTOR inhibitors specifically (Chang and Ganem, 2013). Thus, we hypothesize the role of such modification over total deletion of PTEN as a prime controlling factor of mTOR-targeted therapeutic approach. Successful clinical application of mTOR inhibitors is currently available, such as therapeutic modalities in this case. However, on this note, very little research is available in terms of the role of host–viral genetic interaction responsible for chemotherapeutic resistance or sensitivity of this drug in the case of KSHV malignancy. A clear understanding of chemotherapy-associated host–viral interaction and the genetic or regulatory targets responsible for the therapeutic sensitivity or resistance of a particular drug will make the therapy more specific in terms of its application. Identifying and understanding the sensitivity or resistance determinant as the molecular marker for a therapeutic application needs to be emphasized. In this current study, we tried to analyze the impact of endogenous *wt* PTEN expression in KSHV-infected cells as the principal genetic determinant for induction of cell death by mTOR inhibitors.

On this note, it should be kept in mind that chemotherapeutic drug resistance or sensitivity is a part of the cellular and oncogenic stress management system. The control of balancing cellular and oncogenic stress response decides the fate of a cell post any kind of stress exposure, either therapeutic or growth related, in a tumor microenvironment. When oncogenic stress management dominates, the cancer cell manages to survive the stress. Contradictorily, cellular stress management diverts survival toward death (Liu et al., 2016). In relation to this, in our previous work, we have clearly demonstrated such continuous stress balance inside KSHV-associated malignant cells. mTOR has been reported as a major stress-responsive regulatory pathway (Das et al., 2022). Thus, in this study, we intended to critically intersect the role and regulation of PTEN (the principal mTOR regulator) as an oncogenic stress balancer. Moreover, we specifically tried to concentrate on the impact of the modulated PTEN on making the KSHV-infected cells a stress-modulatory therapeutic target with the application of mTOR inhibitors.

## Materials and methods

### Cell lines and culture maintenance

For the current study, we have used the non-KSHV-infected human embryonic kidney cell lines HEK293 and HEK293T (generously provided by Prof. ErleS Robertson, University of Pennsylvania) that were grown in DMEM (1X GlutaMAX, 10566-016, Gibco, United States) supplemented with 10% serum (FBS, H1 Australian origin, 10100147, Gibco, United States) and 1% antibiotic solution (Pen-Strep, 1140-122, Gibco, United States). KSHV-transformed HEK293T used in the study was generated by stable transformation of *wt* KSHV clone BAC 16 in the laboratory. These cells were maintained in complete DMEM (1X GlutaMAX, 10,566-016, Gibco, United States) supplemented with 50 μg/mL puromycin. The BJAB cell line used in this study as a non-infected (non-KSHV/EBV) B-cell lymphoma (Burkitt’s lymphoma) cell line (Gasperini et al., 2005) was also kindly provided by ErleS Robertson’s laboratory and maintained in RPMI 1640 media (61870-036, Gibco, United States) with 10% serum and 1% antibiotic supplementation (Pen-Strep, 15140-122 Gibco, United States). Different KSHV-positive cell lines, BC-3, HBL-6, and JSC-1, used in this study are B-cell lymphoma cell lines gifted by Prof. David J Blackbourn, University of Birmingham. These cells were cultured in RPMI 1640 media (61870-036, Gibco, United States) with 10% serum (FBS, H1 Australian origin, 10100147, Gibco, United States) and 1% Pen-Strep supplementation (Pen-Strep, 15140-122 Gibco, United States) (Liu et al., 2001). All the cells were maintained in the required aforementioned complete growth media, with or without serum, for the *in vitro* stress-induced model, which has been utilized in our previous work (Das et al., 2022) at 37°C and 5% CO_2_.

### PBMC isolation and maintenance

Whole blood was collected from healthy donors (showing basic health parameters) with the approval of the institutional human ethics committee (VB/IECHR/06/2022; Department of Biotechnology, Visva-Bharati, Santiniketan; 30/06/2022) by taking consent from each individual. In brief, 3 mL of whole blood sample was overlayed on 3 mL of histopacque-1077 (RNBB8622; MilliporeSigma, United Kingdom) followed by centrifugation for 30 min At room temperature at 3,000 rpm, the buffy coat layer containing PBMC was collected (Liu et al., 2021). The collected cells were washed with PBS, and trypan blue exclusion test for cell viability assay was performed (Strober, 2015). PBMCs containing 80%–90% viable cells were maintained in complete growth media containing RPMI 1640 (61,870-036, Gibco, United States) with 10% FBS (FBS, H1 Australian origin, 10100147, Gibco, United States) and 1% penicillin–streptomycin supplementation (Pen-Strep, 15140-122 Gibco, United States) or without FBS (in case of serum-stressed condition) in a CO_2_ incubator maintaining 37°C and 5% CO_2_ levels for 6–24 h depending on experimental need. Post incubation, these cells were processed for immunocytochemistry assays, either for imaging cytometry or flow cytometry. The PBMC population has been selected by bivariate FSC vs. SSC dot plot acquired in a flow cytometer (BD FACSVerse, BD Bioscience) by the BD FACSuite software.

### Plasmid DNA construct details

PTEN clone 1 was purchased from GenScript, USA (Clone ID: M12583), and PTEN clone 2 was provided by Prof. CN Kundu, KITT School of Biotechnology, Bhubaneswar, India; vFLIP K13-GFP and PCDNA were gifted by Dr. Patricia S. Steeg, National Cancer Institute, and Dr. Matthew B Rettig, UCLA School of Medicine, United States. PTEN siRNA was purchased from Invitrogen (4427037 SIL SEL SiRNA INV STD, 1 NM; Assay ID_s534589; 29349990 1).

### Plasmid DNA transformation and isolation

All the plasmids were amplified by transforming into competent *E. coli* DH5α following the Hanahan method (Green and Sambrook, 2018). For the transformation of each set, 1–2 µL of plasmid DNA was transferred into 200 µL of the competent cell contained in a chilled microcentrifuge tube. After proper mixing, the sample was kept in ice for 30 min. Then, the tubes were exposed to heat shock at 42°C in a water bath for 90 min. Post heat exposure, the tubes were immediately transferred to ice and allowed to chill for 1–2 min. Following this, the sample was supplemented with 800 µL of Luria Bartani growth media and incubated at 37°C with continuous shaking for 45 min to 1 h. For selection of the transformed bacterial cells, the bacterial culture harboring the plasmid DNA was spread over nutrient agar plates containing respective resistant antibiotics against the transformed plasmids and grown overnight at 37°C (Green and Sambrook, 2018).

Plasmid DNA from transformed bacterial cells was isolated and purified by using a centrifuge-based filter purification kit, Invitrogen by Life Technologies, USA, PureLink Quick Plasmid DNA Miniprep Kit (Cat. No. K2100-10), and PureLink HiPure Plasmid Filter Maxiprep Kit (Cat. No. K210016) following the manufacturer’s protocol.

### Transfection *via* electroporation and checking transfection efficiency

For transferring the indicated plasmids to the target cell lines, electroporation has been performed using the BioRad Gene Pulser Xcell Electroporation System. For each transfection process, 10 million cells were taken, washed in PBS (ph7.4 (×1) 10010023, Invitrogen), and resuspended in 400 µL of serum-free media (DMEM/RPMI 1640). The resuspended cells were electroporated using 0.4 cm electroporation cuvettes. HEK293/293T cells were transfected at a 210 V, single-pulse, exponential decay protocol, and for BJAB, JSC-1, and BC-3 cell transfection, an exponential decay protocol with a single pulse was followed at 310 V.

For the knockdown assay with PTEN siRNA transfection, the silencer Select Pre-designed siRNA (ID s534589, Ambion by Life Technologies, USA) was used. Transfection was performed by electroporation using a BioRad Gene Pulser Xcell Electroporation system with a liposome-based siRNA delivery system using the Lipofectamine RNAiMAX transfection reagent (Ambion 13778100) by following the manufacturer’s protocol.

All the cells post-transfection were transferred and maintained in their respective complete growth media at 37°C and 5% CO_2_ levels for the required time period (48 h for all the experiments; 24, 48, 72, 96, and 102 h for the time kinetic growth assay).

### Indirect immunostaining for identifying intercellular protein expression and localization

For analyzing the expression of intracellular levels of PTEN, indirect immunofluorescence staining of PTEN was performed. Cells with or without PTEN transfection were washed in PBS, fixed with pre-chilled 1:1 acetone methanol solution following the PBS wash, and permeabilized with 1% triton X100 (MB031, Himedia, India). Then, the cells were subjected to PBS washing (at 14,000 rpm, 5 min at room temperature) and blocked with 1% BSA (MB 083, Himedia, India) in PBS/TBS buffer overnight at 4°C. Post-blocking, cells were further washed with PBS and incubated with 1:100 dilution of rabbit anti-PTEN-primary antibody (CST 138G6, United States) for 1 h at room temperature. Following primary antibody incubation, cells were subjected to PBS wash, fluorescent-tagged secondary antibody, and anti-rabbit FITC (BD Bioscience, 554020, United States) and incubated for 1 h at room temperature. Then, the cells were washed again with PBS and were acquired in a flow cytometer (BD FACSVerse, BD Bioscience, United States) at their respective channel and analyzed using the BD FACSuite software by Biosciences, United States, version 1.0.6.

Imaging cytometry was performed for localization and colocalization studies for analyzing the nuclear–cytoplasmic partitioning of proteins that were grown (for HEK293/293T only) or dried over a poly-L-lysin (sigma)-coated coverslip (for KSHV-infected suspension cells). Following this, the aforementioned steps were performed, starting from fixation to secondary antibody incubation. Last, for nuclear staining, cells were incubated with DAPI post antibody incubation in PBS for 3–5 min at RT and washed with PBS. The stained cells in the coverslip were mounted over glass slides using the ProLong Gold Antifade reagent and analyzed under a BioRad Zoe fluorescent cell imager or fluorescent microscope (BioRaD, USA). For colocalization study of different proteins, the primary antibody of rabbit anti-PTEN (CST 138G6, 1:100 dilution) with the respective secondary antibody tagged with fluorochrome was used (Anti-Rabbit Dylight 549, BioRad, United States, at 1:50 dilution) detected in the red channel; intracellular vFLIP-GFP detection was performed in the green channel. Merged images for those respective channels were taken for analysis by using a BioRad Zoe fluorescent cell imager or ImageJ software (version 1.8.0, NIH, United States). Image compilation with acceptable adjustment was performed for better representation using ImageJ software.

### Cell viability assay

KSHV-infected PEL cells were subjected to propidium iodide cell viability assay post different treatment conditions and PTEN overexpression at different doses. 1 × 10^6^ cells for each set were given treatment and incubated for the required time period with or without treatment. Then, those cells were washed with PBS and incubated with 50 μg/mL propidium iodide (PI) at room temperature for 15 min and analyzed and acquired by flow cytometry (BD FACSVerse). The percentage of PI-positive cells inserted within the cell with loss of membrane integrity and viability, compared to PI-refused non-transfected cells and transfected empty vector as control with high viability, was measured in BD FACSuite software (BD Biosciences, United States, version 1.0.6). Moreover, cell death as a consequence of overexpression of PTEN or empty vector was measured by PI vs. FITC dual-stain assay, where indirect immunostaining of intercellular PTEN was performed. PTEN overexpression was measured by the positive fluorescence FITC channel (described earlier under indirect immunostaining, in materials and methods ), and its associated cell death was assessed by measuring the fluorescence intensity of PI channel acquired in BD FACS VERSE. Fluoresence-positive and -negative population percentages were calculated in BD FACSuite software (BD Biosciences, USA, version 1.0.6).

### Annexin V-FITC assay for apoptosis

For analyzing the apoptotic events, 1 × 10^6^ cells were seeded post-PTEN and empty vector transfection along with different mTOR inhibitory drug-treated sets were washed with PBS and subsequently processed for analyzing with FITC Annexin V Apoptosis Detection Kit II (BD Pharmingen, 556570, United States) based on analyzing apoptotic cells, characterized with altered membrane integrity. According to the manufacturer’s protocol, the PBS-washed cells (1 × 10^6^ cells) were resuspended with ×1 binding buffer. Following this, the cells of each experimental sets were incubated and processed for staining with FITC + PI, only FITC, only PI, and unstained control sets each having 1×10^5^ density. Then, the cells were acquired in BD FACSVerse, and data were analyzed in BD FACSuite software (version 1.0.6, BD Biosciences, United States) and represented with FITC vs. PI dual-stained densitometric quadrate plots. Apoptosis is characterized by loss of plasma membrane with externalization of membrane-bound phosphatidylserine (PS). Phospholipid binding protein Annexin V shows high affinity toward PS, and thus, Annexin V-FITC positivity indicates early apoptosis. To clarify the exact stage of the apoptosis, this assay is combined with another DNA-binding dye, propidium iodide (PI), indicating cell viability. A viable cell with an intact membrane will exclude the entry of PI into the cell, whereas as the cell loses its viability due to programmed cell death, it will allow PI to enter inside. Thus, Annexine V-FITC and PI dual-positive cells indicate late-stage apoptosis and Annexin V-FITC-positive PI-negative cells are late-stage apoptotic and necrotic cells (Vermes et al., 1995).

### Analyzing mitochondrial membrane potential

PTEN-overexpressed and silenced conditioned KSHV-infected cells were subjected for analyses of mitochondrial membrane potential using a flow cytometry-based detection kit BD MitoScreen, JC-1 Kit, (55132, BD Biosciences, United States). The treated cells post incubation were subjected for PBS wash and processed for JC-1 staining by following the manufacturer’s protocol**.** Data acquisition was performed in BD FACSVerse and analyzed in BD FACSuite. Mitochondrial membrane potential is measured by the JC-1 membrane permeable fluorochrome status in aggregates or monomeric conditions. Healthy cells with polarized mitochondria uptake the JSC-1 from the cytoplasm, which forms aggregates inside, emitting red spectra, whereas apoptotic cells, with depolarized mitochondria, cannot hold JC-1 aggregates, thus pumping it back to the cytoplasm, with its monomeric form emitting green spectra. Thus, the red vs. green bivariate plot with a high cell percentage in the red spectral zone indicated a healthy cell with polarized mitochondria, and more cell percentage in the green zone indicated damaged and depolarized mitochondria (Cossarizza et al., 1993).

### Cell cycle detection

Different stages of the cell cycle in the PTEN-overexpressed and silenced KSHV-infected stress-induced *in vitro* cell line model were subjected to analysis based on nuclear DNA content using propidium iodide. A dividing cell consists of several cell cycle phases, mainly categorized into interphase (the phase between cell division) and mitosis (the divisional phase). During these phases, the DNA distribution varies, depending on the function of each stage. During interphase, G0 is the phase that determines whether a cell undergoes division. Following the S phase, the DNA continues to replicate, and in the next G2 phase, it allows the cell to multiply its DNA before entering into the mitosis phase (M phase). PI-based flow cytometric assay can quantify the proportion of cells in each phase of the cell cycle based on DNA content. For this analysis, the cells were washed with PBS and fixed in chilled 70% EtOH in ice for 15–30 min. Following fixation, the cells were again washed with PBS and stained with 10 μg/mL PI in TritonX 100 buffer containing RNase and incubated for 30 min at room temperature. Processed cells were acquired in BD FACSVerse (BD Biosciences, United States). The percentage of cell population in each state of the cell cycle (Sub G0/G1; Go/G1; S; G2/M) was analyzed from the PI vs. cell count plots based on the DNA contents in BD FACSuite software (version 1.0.6, BD Biosciences, United States).

### Western blot

Alterations in the intercellular protein levels inside KSHV-infected cells with dose-dependent mTOR inhibitor treatment were analyzed by Western blotting. Post treatment, the cells were lysed in radioimmunoprecipitation assay buffer (RIPA) containing protease inhibitor cocktail (P8340, MilliporeSigma, United States). The protein lysate was mixed with loading buffer and heated for 3–5 min at 95°C. An equal amount (20 µg) of extracted protein from treated and control sets was loaded for each lane and subjected to separation by 12% SDS-PAGE electrophoresis. Following this, the gel is electrotransferred to a nitrocellulose membrane and blocked for 1 h at room temperature with 5% skimmed milk in tris base saline Tween-20 (TBST). After blocking, the blots were then probed with specific primary antibodies and incubated at 4°C overnight. Following the primary antibody incubation, the blots were subjected to TBST wash and HRP-tagged secondary antibody incubation (specific against respective primary antibody) for at least 1 h at room temperature. After proper incubation, the membrane was washed well with TBST and visualized in the Chemidoc XRS + image system equipped with ImageLab software (BioRad, United States, Version 5.2) using the BioRad ECL chemiluminescent substrate. The intensity of the protein band was analyzed and quantified in ImageJ software (by NIH, Version 1.8.0). The value is normalized with loading control (GAPDH) and finally with non-treated control. Primary antibodies of rabbit anti-PTEN (CST 138G6), rabbit anti-p-PTEN(CST, 9551), and mouse anti-p53 (CST, 1C121:1,000 dilution) were obtained from CST, United States of America. Mouse anti-ATM (Sc 377,293), mouse p-ATM (SC 47739), mouse anti-Chk1(SC 8408), mouse anti-Chk2 (sc-5278), rabbit anti-p-Chk (sc-101658), GAPDH (mouse mAb cat. No. sc-166545), and mouse anti-cMyc (sc-9E10) were purchased from Santa Cruz Biotechnology, United States. All primary antibodies were used at 1:1,000 dilution. Anti-mouse (11-301, Abgenex, India) and anti-rabbit (ABclonal Biotech, United States, AS014) HRP-tagged secondary antibodies were used at 1:5,000 dilution.

### ROS vs. PI assay

Oxidative stress induction and its associated cell death in the PTEN transfected or non-transfected KSHV-infected stress-induced *in vitro* cell line model has been analyzed by the DCFH-DA vs. PI dual staining procedure. For this analysis, PTEN transfected/non-transfected cells (1 × 10 ^6^ cells per set) were maintained in normal growth conditions following the previous transfection protocol for 48 h. Post incubation, these cells were exposed for, with or without serum stress, 24 h (following the previously described serum stress-induced *in vitro* model). Subsequently, post stress incubation, they were revived with normal growth conditions for 24 h and subjected to ROS vs. PI assay. For this analysis, these cells were washed in PBS and resuspended in serum-free RPMI media with 50 µM of DCFH-DA (Sigma-Aldrich, 2′,7′- dichlorofluoresin diacetate, D6883) for 30 min at 37°C in the dark. Following PBS wash, these cells were processed for PI staining for cell viability assay (as previously mentioned). PI-stained cells were washed in PBS and flow cytometrically analyzed. The intracellular ROS was determined by analyzing the fluorescence intensity of DCFH-DA at 488-nm excitation and 530-nm emissions (FITC channel); cell death was measured by PI intensity. The further quantification of population percentage was determined by FITC VS. PI quadrate plots by flow cytometry analysis (BD FACSVerse, using BD FACSuite software, version 1.0.6, BD Biosciences, USA).

### Autophagy vs. PI assay

Intracellular autophagy induction was measured by MDC staining in association with PI cell viability assay. Following transfection and growth in normal culture condition for 48 h., the KSHV-infected cells with/without PTEN overexpression were maintained following the serum stress-induced model, as previously described. These stress-exposed with/without PTEN transfected cells were then stained with 50 µM MDC/dansylcadaverine (Sigma-Aldrich, BCBX0277) for 30 min following PBS washing and PI staining for viability check. Post staining, the cells were again washed and resuspended in PBS, and the percentage of cell population with their fluorescence intensity were measured by flow cytometry (BD FACSVerse) in a V500 channel at 500 nM of wavelength (for MDC) and a PI channel at 585 nM (for cell death). Data analysis has been performed in BD FACSuite software (version 1.0.6, BD Biosciences, United States).

Similarly, PTEN transfected/non-transfected KSHV-infected cells were exposed with/without mTOR inhibitor-induced stress at their respective doses, following the serum stress-induced model. These cells were processed for MDC vs. PI assay and analyzed by flow cytometry as described earlier.

For detecting specific autophagic involvement, PTEN transfected/non-transfected/silenced cells were grown in the presence/absence of an autophagy inhibitor, bafilomycin-A1 (B1793, MilliporeSigma, USA), at 20 nM working condition, post serum stress, or mTOR inhibitor stress exposure process, during the recovery period.

### Statistical analysis

Statistical analyses were performed in GraphPad Prism (version 6). Average data obtained from triplicate values for each independent experiment were repeated three times. An unpaired *t*-test and one-way ANOVA analyzed the difference between the means of the two groups. Two-way ANOVA was followed by Bonferroni’s post-test, which was performed to assess significance, in comparison between more than two groups. The statistical values in different experiments represent the mean of ±SD where n equals to 3. A probability (p) value of <0.05 was considered as significant. *- *p* ≤ 0.05, **- *p* ≤ 0.005, and ***- *p* ≤ 0.0001, and NS (non-significant) denote the statistical significance level of the data.

## Results

### Endogenous expression of PTEN and its effect on cytotoxicity due to overexpression in KSHV-infected cell lines

**FIGURE 1:**
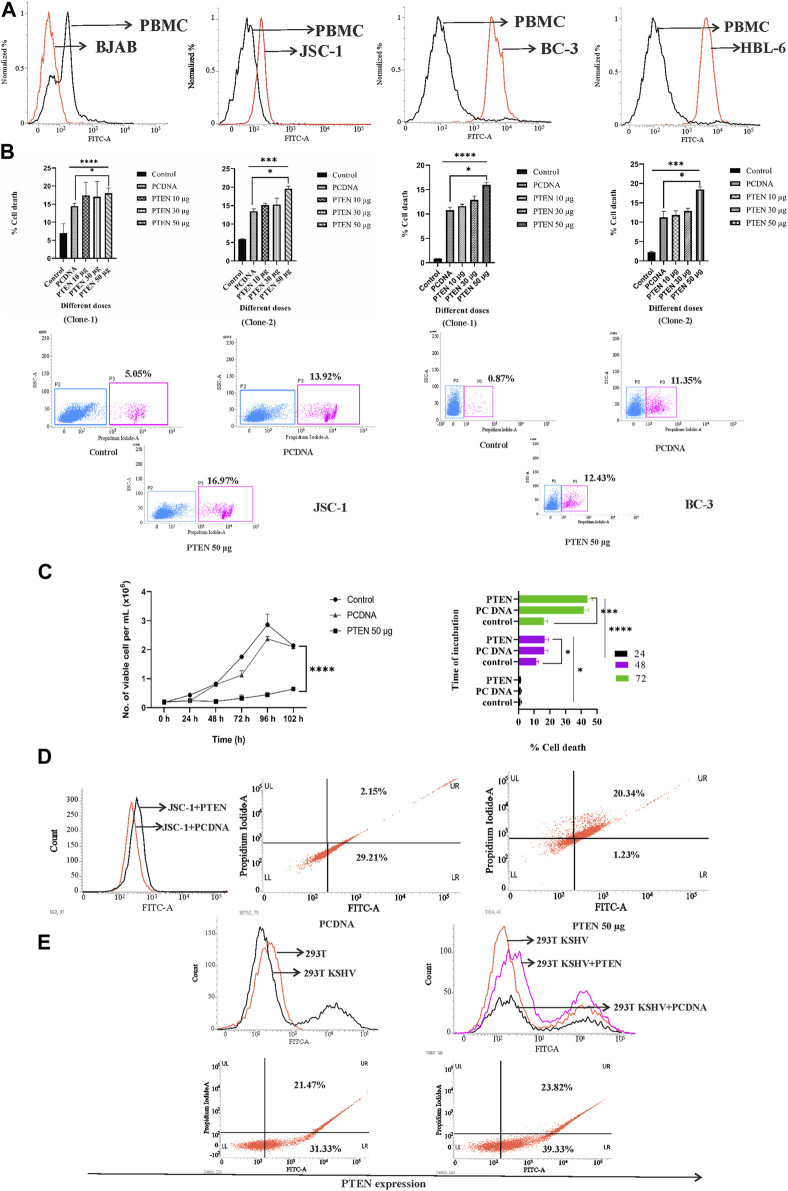
Assessment of endogenous PTEN and the effect of PTEN overexpression against KSHV-infected cells. **(A)** Histogram overlay representing the comparative endogenous PTEN status, represented by normalized fluorescence intensity and measured by the FITC channel. **(B)** Bar charts showing statistically analyzed dead cell percentages post dose-dependent PTEN overexpression against different KSHV-infected cells. Representative dot plots have been presented with PI-positive cell population percentage. **(C)** Line diagram representing time kinetic cell growth post-PTEN overexpression. Bar chart indicates the comparative cell viability in time kinetic manner in different treatment sets. **(D)** Histogram overlay showing transfection in KSHV-infected cell efficiency by intercellular PTEN expression represented in the FITC channel. Dot plots show endogenous PTEN expression (FITC channel) vs. cell death (PI channel). **(E)** Histogram overlays representing intercellular PTEN level and transfection efficiency in transformed KSHV HEK293T cells. Dot plots show PTEN expression (FITC channel) vs. PI cell percentage for the same.

For investigating the variation of PTEN expression compared to healthy cells, we intended to check the endogenous PTEN level in isolated PBMCs and compared it with different KSHV-infected cells (JSC-1, BC-3, and HBL-6) by immunofluorescence assay. Following the previous finding, the comparative histogram plot analysis (shifting of representative histograms indicating PTEN expression with FITC fluorescence signal) clearly reveals that all the KSHV-infected cell lines express a highly constitutive intercellular PTEN protein level compared to human PBMC. Here, we have represented the endogenous PTEN expression plot in the BJAB cell line as a negative control to set the basal level (cut off signal) for histogram shifting analysis. BJAB is a non-infected B-cell lymphoma cell line which harbors the totally deleted PTEN gene (Figure 1A). Our results strongly indicate the fact that KSHV-infected malignant cells overexpress the native form of PTEN, but despite that, as it is hyperphosphorylated, so its functionality is utilized for the sake of the pathogen for the malignancy maintenance process.

To further analyze the functional importance of this wild type native form of PTEN expression, we further overexpressed the intact PTEN gene exogenously by inserting different full-length PTEN clones (PTEN-1 and PTEN-2) in KSHV-infected cells by electroporation. A dose-dependent assay (10, 30, and 50 µg) with two different PTEN clones has been performed against two different KSHV-infected cell lines (BC-3 and JSC-1) for 24 h of incubation post-transfection. Comparative bar diagrams of PI-positive cell percentage along with representative dot plots shows that all the PTEN transfected sets showed significant induction of loss of cell viability with increased number of PI-positive cell population, allowing the dye to pass inside with their degraded membrane compared to non-transfected control. If we consider the comparison with the empty vector control set (PCDNA), only 50 µg of the PTEN transfected set showed a little hike in its significance in terms of the viability loss (Figure 1B). From these results, we have analyzed that both BC-3 and JSC-1 cells followed the same pattern of PTEN-mediated cell viability loss with both of the full length PTEN clone (Figure 1B left and right panels).

Next, we intended to perform a time kinetic cell growth (with KSHV-infected cell JSC-1) analysis at the representative effective dose of PTEN treatment (50 µg). From this analysis, we actually tried to figure out how the PTEN-mediated cell viability loosing process undergoes inside a KSHV-infected cell. We incubated the PTEN transfected KSHV cells for a prolonged period of time post-transfection, and we checked the cell viability percentage compared to empty vector (PCDNA) at every 24 h time interval. The results at each time point showed that KSHV-positive cells start losing the viability process compared to empty vector and non-PTEN-overexpressed control cells after 24 h post-transfection (Figure 1C, left panel). Figure 1C, right panel, graphically representing the time kinetic differential cell death percentage with respect to the empty vector which shows that the process gradually reaches the increased significant level starting post 48 h till 102 h post-transfection**.**

Next, to check whether the loss of viability event is specifically a result of PTEN overexpression or not, we performed an assessment of PI cell viability in accordance with PTEN overexpression confirmation. For this, the KSHV-infected cells were transfected with PTEN and its empty vector PCDNA. After these transfected cells were incubated for 48 h (showing significant viability loss previously), they were first stained with PI for viability assay and then processed for immunostaining to detect the variation of endogenous expression level of PTEN compared to PCDNA. To avoid non-specific PI insert due to the permeabilization step of immunostaining, PI staining was performed before the immunostaining process. The results clearly showed that enhanced endogenous PTEN overexpression is associated with lack of cell viability (FITC-PI dual positive cells) in PTEN transfected cells, whereas the empty vector-transfected cells showed KSHV cell population distribution at only FITC-positive block in the FITC-PI dual quadrate plots, with a very little amount of dead cells represented with PI-FITC dual positivity. These results confirmed the fact that the reduced viability is due to the effect of the exogenous PTEN delivery in the KSHV-infected cell line (Figure 1D).

We have also checked the effect of PTEN overexpression in KSHV-transformed stable cell line HEK293T by PI vs. PTEN immunostaining assay. The results clearly depict the low level of PTEN expression in KSHV-infected HEK293T cells compared to normal HEK293T cells, which confirms the fact that KSHV infection lowers the PTEN gene expression inside cells. Moreover, the overexpression of PTEN in the transformed HEK293T KSHV cell (Figure 1E, upper panel) can also successfully induce the loss of viability process compared to PCDNA (Figure 1E, lower panel).

### KSHV-infected B lymphoma cell lines are sensitive to PTEN-induced programmed cell death process

**FIGURE 2:**
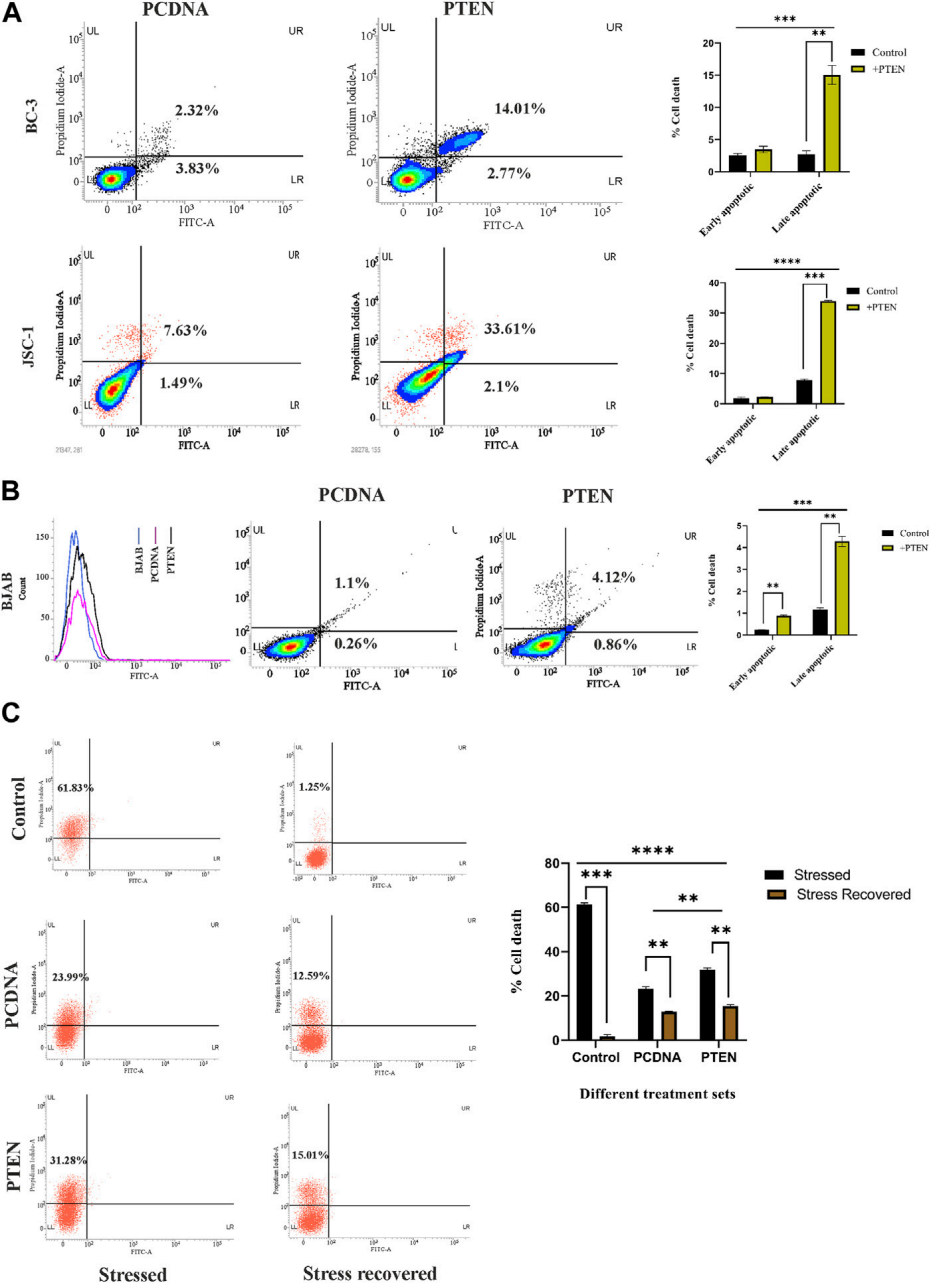
Role of PTEN in apoptotic events inside KSHV-infected cells. **(A)** Densitometry plots and bar charts representing different stages of apoptotic cell death events in different KSHV-infected cells with PTEN/PCDNA transfected conditions. **(B)** Comparative histogram overlay plot represents transfection efficiency of PTEN in the PTEN-deleted BJAB cell line. The densitometry plots with bar charts shows the Annexin V-FITC assay results of indicating apoptosis. **(C)** Annexin V-FITC dot plots, along with a comparative bar chart in the *in vitro* stress-induced KSHV cell line model with PTEN/PCDNA transfected and non-transfected control sets.

Following the aforementioned findings on PTEN expression in KSHV-infected cells**,** we further investigated the in-depth process, prior to viability loss, to clarify the mode of PTEN-mediated induction of a cell death process. For this, we analyzed whether the externally induced PTEN gene can initiate any programmed cell death event in these cells. For this, we performed an Annexin V-based apoptosis assay, which can detect the apoptosis process in viable cells (as pre cell death event/early-stage apoptosis) and late-stage apoptosis with loss of viability (late-stage apoptosis) along with total cell death (necrosis). Referring to this, we have transfected two different PTEN plasmids at their effective doses (50 µg) against KSHV cells to increase the level of total intact PTEN protein. These transfected cells were incubated for 48 h post-transfection for exerting PTEN efficacy on initiating the process of effective viability loss, and the mode of PTEN-mediated cell death process was analyzed during this period (Figure 2A). The PI vs. Annexin V-FITC plots in PTEN transfected vs. PCDNA transfected sets clearly show that PTEN effectively induces apoptosis in both BC-3 and JSC-1 cells. This result suggests that KSHV-infected cells lose their viability due to the exogenous PTEN-induced programmed cell death process.

Furthermore, to critically analyze the role of PTEN-mediated cell death initiation in B-cell lymphoma, irrespective of the infectious condition, we have tested the efficacy of PTEN against a non-infected B-cell lymphoma cell line, BJAB, where PTEN is totally deleted with KSHV-infected B-cell lymphoma lines. The effect of PTEN overexpression has been compared with the PTEN null non-infected B-cell lymphoma cell line, BJAB, and PTEN-mutated KSHV-infected B-cell lymphoma cell lines. The percentage of apoptotic cells was found to increase post-PTEN transfection in both KSHV-infected and non-infected B-cell lymphoma, but KSHV-infected cells were found to be more sensitive against PTEN overexpression in terms of the number of death percentage. Moreover, this result, in association with PTEN status variation between the two cells, indicates the fact that the presence of endogenous PTEN in the infected cells makes them more sensitive against the overexpression compared to the total deleted condition (Figure 2B).

These cell death-related assays strongly establish the fact that PTEN modulation is reversible and the PTEN-mediated apoptosis process is active inside KSHV-infected cells lines. Now, the question is what is the role of this intact functionality of PTEN inside KSHV-infected malignant cells? To reveal the fact, we have checked the performance of PTEN under a conditional environment with serum stress in our next set of experiments. For this, we have checked the role of the PTEN-induced cell death process in the stress-exposed *in vitro* cell line model. The results interestingly showed that PTEN can lower the serum starvation-induced programed cell death process in KSHV-infected cells compared to control (Figure 2C, left panel). Moreover, it is also observed that the rate of the starvation recovery process is high, and stressed cells are totally recovered by the normal growth condition, whereas in the presence of PTEN, it is not possible (Figure 2C, right panel). Collectively, these data suggest that under stress, PTEN acts as stress balancer and under normal conditions, it acts as a death inducer in KSHV-infected cells.

### Differentially expressed PTEN acts a stress balancer in response to stress inside KSHV-infected cells

**FIGURE 3:**
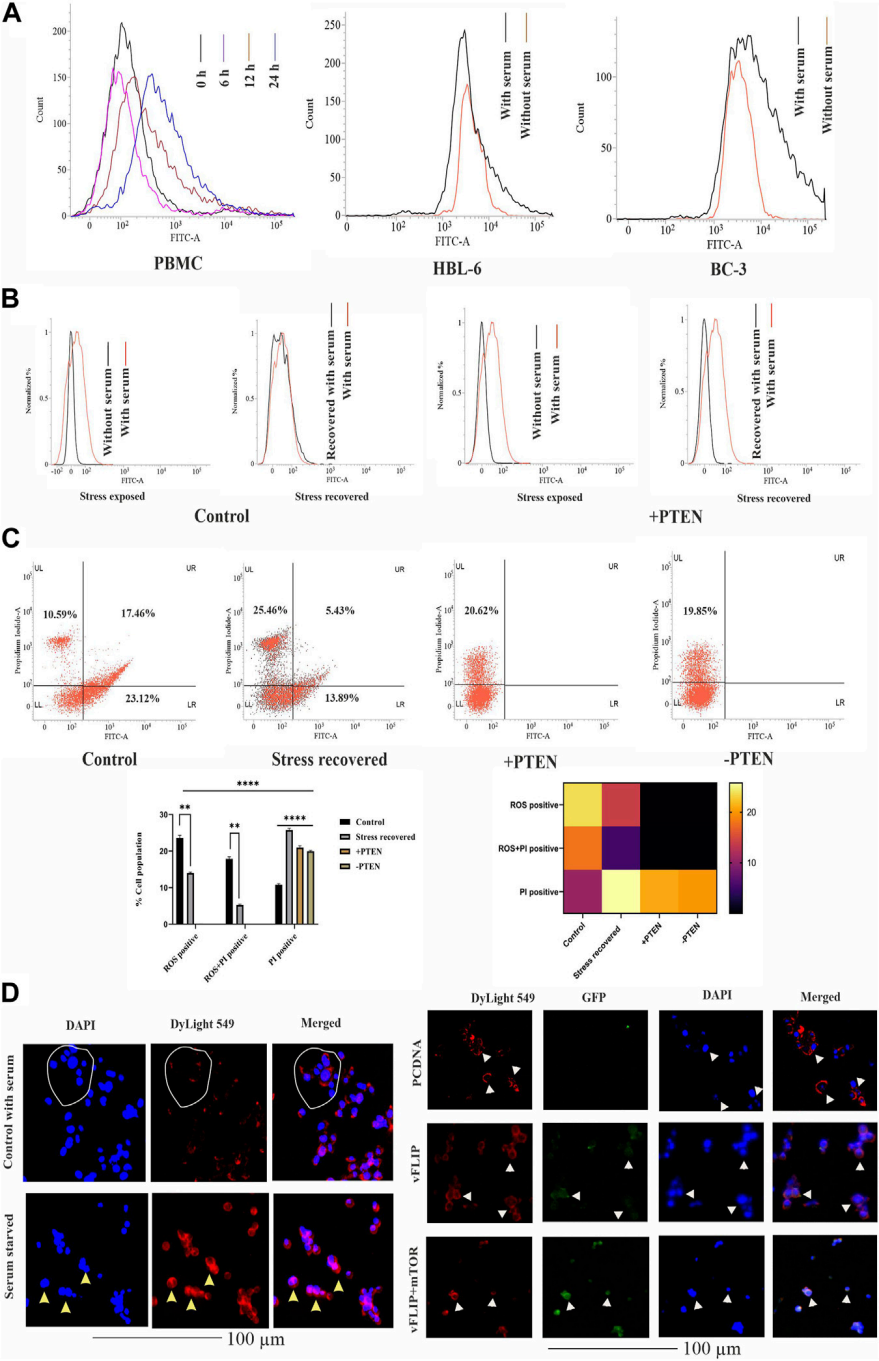
Effect of PTEN as a stress-sensitized molecule in normal and KSHV-infected cells. **(A)** PTEN immunofluorescence intensity has been represented with concomitant serum stress exposure in PBMC and KSHV-infected cell lines. **(B)** Intracellular ROS level in the stress-induced *in vitro* cell line model in KSHV-infected cells post-PTEN overexpression compared with control. FITC channel represents the ROS signal. **(C)** Dot plots illustrating ROS vs. PI alterations in KSHV-infected cells with or without PTEN overexpression. Bar diagram states comparative cell population in each block of the dot plot. Heat map presentation demonstrates the densitometric changeover of the SEM value of each population. **(D)** Left panel images represents the localization of intracellular PTEN in 293 cells in normal and serum starved conditions, where DyLight 549 indicates PTEN fluorescence. White circles and yellow triangles represent the identified cells. Right panel images are microscopic observation of vFLIP–PTEN interaction. vFLIP expression has been represented by GFP fluorescence and intracellular PTEN represented by DyLight 549. White triangles indicate the representable cells.

Next, we investigated the stress-induced PTEN expression pattern inside KSHV-infected cells. For this, we utilized serum stress as a conditional factor to track the functional direction of PTEN in KSHV-infected cells. Here, we first targeted to detect the pattern of expression of PTEN in healthy PBMCs in a differential nutritional condition to check whether KSHV-infected B cells follow the same path. Here, cells were exposed to serum stress for 6–24 h, and the expression level of endogenous PTEN has been checked by indirect immunofluorescence study using flow cytometry. The time kinetic histogram overlay plot of both serum stress and stress revived condition clearly shows that PTEN is overexpressed inside PBMCs in response to serum stress. Then, similarly, KSHV-infected cells (HBL-6 and BC-3) were exposed to 24 h of serum starvation, and an increased endogenous PTEN level was observed alike PBMCs (Figure 3A). This result clarifies that serum stress acts as a conditional factor for differential endogenous PTEN expression, both in healthy and KSHV-infected B cells.

So, next, we have analyzed the role of stress-responsive PTEN in regulation of the KSHV stress management system in the *in vitro* stress-induced model. Here, a comparative histogram analysis has been performed to check the intercellular ROS level in stress-exposed and stress-recovered conditions with or without PTEN overexpression. Interestingly, it is observed from the result that KSHV-infected cells maintain an enhanced intercellular ROS level, which is narrowed down post serum stress. When the stress-exposed cells are recovered with complete growth media supplemented with serum, these cells again reverted back with similar level of ROS like a normal growing KSHV-infected cell in media. The histogram overlay of the serum-stressed cell has been narrowed and redistributed in recovered cells compared to normal growing cells (Figure 3B, left panel). But the same conditional stress response by the virus can not be managed in PTEN overexpressed cells (Figure 3B, right panel). PI vs. ROS plot assay in PTEN transfected and non-transfected conditions enhances the aforestated finding with a bonus finding of the role of PTEN in KSHV exerted oncogenic stress balance tactics (Figure 3C). Here, the representative dot plots indicates that an enhancer ROS and stress-sensitized cell death process is reactive inside KSHV-infected cells (control vs. stress recovered plot), where PTEN overexpression enhances this stress-induced cell death process, which is not managed by the recovery process. Moreover, PTEN silencing hampers the stress-sensitized death process (+PTEN vs. -PTEN plot). Moreover, the graphical representation of the ROS vs. PI assay clearly demonstrates that the ROS-positive population is decreased in the stress-recovered set compared to normal KSHV-infected cells growing in normal condition, which is totally absent in PTEN-positive and PTEN-negative sets. PTEN overexpression induces the cell death process. Similarly, the ROS–PI-positive population (ROS-associated dead population) has been observed as decreasing compared to control. On this note, it is observed that the PI-positive population is significantly increased in all the three sets compared to control (Figure 3C, lower panel). These results indicate that KSHV-infected cells possess an increased intercellular ROS, and thus, these cells are sensitive to the ROS-mediated cell death process. Enhanced ROS-positive PI population to PI positivity indicates that during the cell’s stress recovery process, this mechanism is enhanced, which might be the cell survival tactics, and the similar target is followed by PTEN more profoundly, which leads to shifting of total cellular population distribution in the PI-positive zone. This stress-sensitized death process is hampered in PTEN-silent cells, confirming that the process is totally PTEN mediated.

It is reported that nuclear and cytoplasmic partitioning of PTEN is important for its functional activity, which is stress induced. So, as our PTEN has been found as a member of the cellular and oncogenic stress balancing mechanism, we intended to check its distribution pattern in both non-infected and in the presence of the KSHV agent. So, first, we checked the pattern of PTEN localization in normal and serum stress conditions in non-KSHV-infected healthy cell line HEK293 as control (Figure 3D, left panel). It is well observed from the localization study that PTEN normally resides at the cytoplasm indicated cells are represented with white circle, and post serum stress, it tends to relocate to the nucleus yellow triangle, which probably indicates its stress-induced gaining of functionality to control gene expression. Thus, we can speculate from this analysis that nuclear–cytoplasmic distribution of PTEN is stress responsive in normal condition. Next, we have introduced KSHV vFLIP to these normal cells and checked the distribution pattern of PTEN inside (HEK293T for better transfection). In this study, we tried to observe the pattern for PTEN-vFLIP distribution to check whether the KSHV agent vFLIP will have any impact over PTEN distribution in an infected condition. The result shows that vFLIP and PTEN mainly tend to localize in the cytoplasm, and immunofluorescence analysis from merged image indicates that both vFLIP and PTEN co-localize in the cytoplasm. The PTEN expression and localization pattern remains intact inside normal indicated with white circles and white triangles and vFLIP transfected HEK293 cells, but the presence of vFLIP might interact with PTEN, which was absent in non-vFLIP transfected cells (Figure 3D, right panel). To further check the functional importance of this interaction and vFLIP-mediated PTEN regulation, we have implied mTOR inhibitor against vFLIP–PTEN transfected HEK293T cells. Our results clearly show that mTOR inhibitors induce a cytotoxic effect, specifically against the vFLIP–PTEN transfected cells, and hit the KSHV–FLIP-mediated oncogenic regulation of stabilized cytoplasmic partitioning of *wt* PTEN and induce cell death (Figure 3D, right panel). It is evident from the fluorescent images that, in the empty vector-transfected sets, the cytoplasmic fluorescence intensity in the merged images is separately distributed, whereas in vFLIP transfected sets, it seems more intense and composite, which indicates vFLIP–PTEN colocalization. Subsequently, with mTOR inhibitor treatment, this pattern tends to destabilize as the fluorescence intensity seems to fade out.

### Endogenous PTEN status determines mTOR drug sensitivity in KSHV-infected cells

**FIGURE 4:**
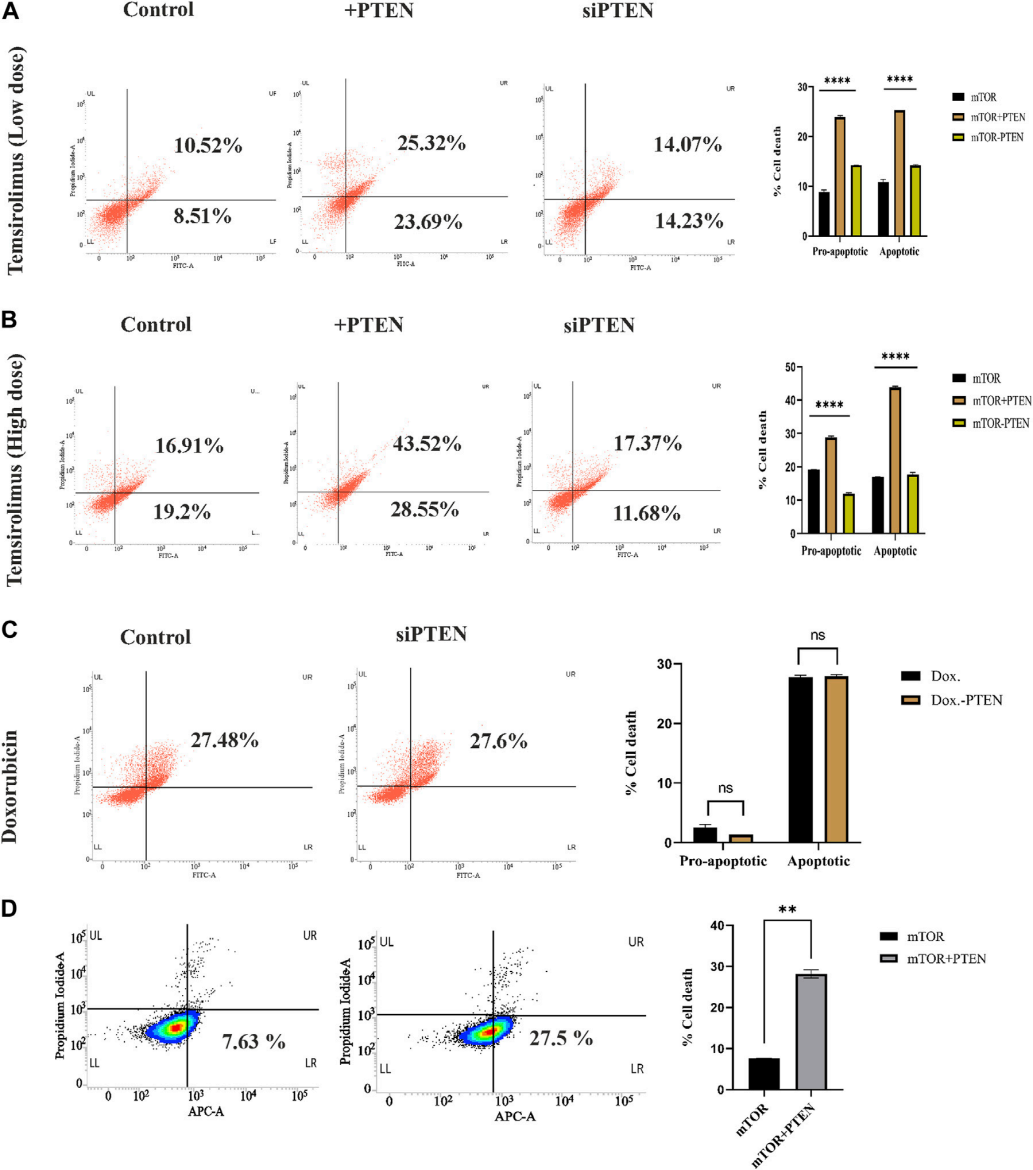
Role of endogenous PTEN status in mTOR drug sensitivity by Annexin V-FITC assay. **(A)** Low-dose mTOR inhibitory effect (low-dose drug-induced stress) in PTEN transfected and silenced conditions compared to non-transfected control against KSHV-infected cells. **(B)** High-dose mTOR inhibitory effect (high-dose drug-induced stress) in PTEN transfected and silenced conditions compared to non-transfected control. **(C)** Doxorubicin efficacy in PTEN-silenced condition. **(D)** Effect of PTEN overexpression with high-dose mTOR drug efficacy on the alteration of an apoptotic event compared to the PTEN-deleted normal condition in BJAB cells.

In the current experiments, we tried to understand whether the constitutive endogenous PTEN expression inside KSHV-infected B-cell lymphoma puts any differential mTOR inhibitor’s sensitivity. Here, we have tried to correlate endogenous PTEN’s functional importance over the mTOR inhibitor’s sensitivity in KSHV-infected cells. More specifically, we intended to investigate whether *wt* PTEN is the major determining regulator of mTOR-induced cell death in KSHV-infected cells. For this, we have analyzed the sensitivity of first-generation mTOR inhibitor temsirolimus against KSHV-infected B cells. We have implied different doses of temsirolimus against the infected cells, to reveal how KSHV-infected cells respond by the PTEN-mediated cell death process in response to handle mTOR inhibitor-mediated therapeutic stress. Post 48 h, PTEN transfected and PTEN-silent KSHV-infected cells were incubated with different doses (low dose at 5 μM; high dose at 20 µM) of temsirolimus and assessed for apoptosis by flow cytometry (Figures 4A, B). The representative plots clearly reveal that at lower dose of mTOR inhibitor, PTEN overexpression can induce the number of cells undergoing apoptosis, where silencing of PTEN hampers the process (Figure 4A). The similar pattern of PTEN activity over mTOR inhibitor sensitivity has been observed at a higher dose in a more profound manner in terms of increased apoptotic cell percentage compared to non-PTEN transfected control (Figure 4B). Moreover, the silencing effect of PTEN has been observed in reducing the enhanced effect of the PTEN-mediated mTOR inhibitor’s drug sensitivity.

Furthermore, to clarify the hypothesis of whether the PTEN status responsible for determining the enhanced sensitivity of mTOR drug also serves as a sensitivity determining factor for other chemotherapeutic agent or not, we have checked the efficacy of a common chemotherapeutic agent, doxorubicin, against these cells, It has been observed that PTEN silencing does not affected the efficacy of doxorubicin, as no significant change in cell death percentage has been observed between these two treatment sets (Figure 4C). This result strongly supports that PTEN is specifically effective in determining mTOR inhibitory drug sensitivity.

We further checked the importance of PTEN status in determining the mTOR inhibitor resistance by comparing the effect of mTOR inhibitors against PTEN-deleted B-cell lymphoma cells (BJAB) and checked the ability of mTOR inhibitors as the modulator of drug resistance in such PTEN-deleted condition. These cells showed low sensitivity against mTOR inhibitors due to the absence of PTEN, and PTEN overexpression enhanced the efficacy of these drugs (Figure 4D). This finding more specifically establishes the fact that PTEN specificity enhances the efficacy of mTOR inhibitors, which is not limited to PTEN-modulated condition, but it also actively works in the PTEN-deleted condition.

### Analyzing the death axis of stress regulator PTEN in KSHV-infected cells

**FIGURE 5:**
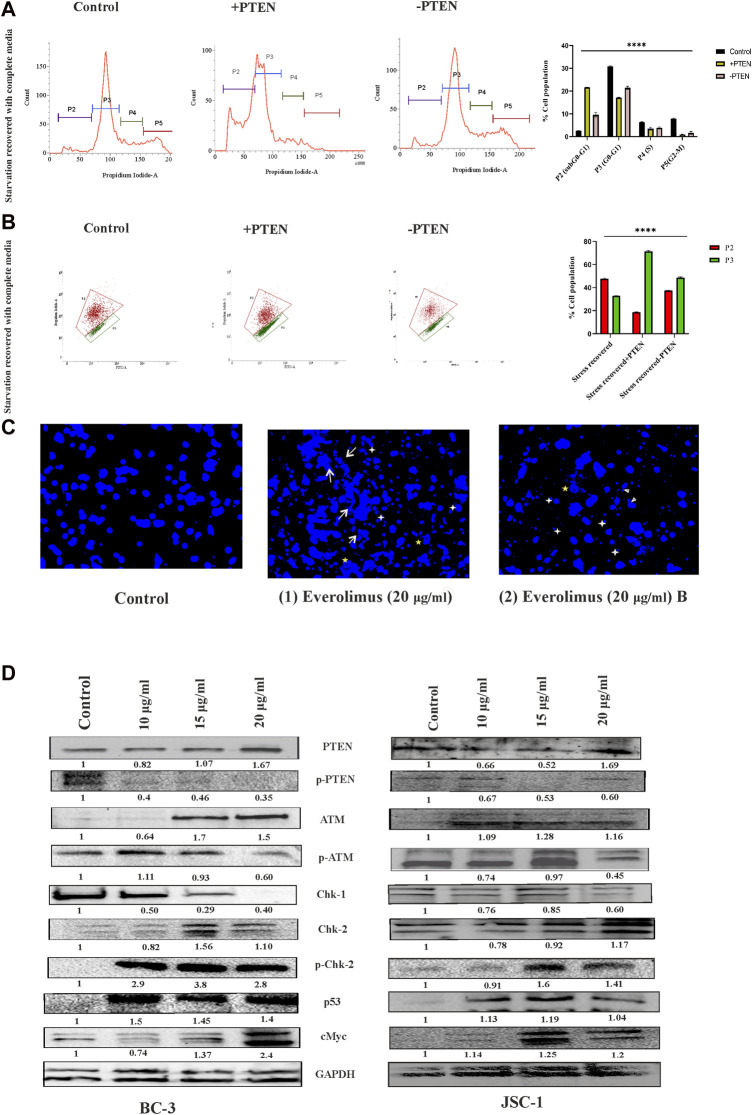
Regulatory death axis of mTOR inhibitors through PTEN in KSHV-infected cells. **(A)** Alterations in cell cycle phases with PTEN transfected and silenced conditions in comparison with non-transfected conditions in the serum stress-induced *in vitro* model. **(B)** Changeover in mitochondrial membrane potential in PTEN transfected and silenced conditions compared to non-transfected control. **(C)** Microscopic images analyzing DNA damage activity by DAPI staining post mTOR inhibitor treatment. Two different everolimus (20 μg/mL) that are treated, Figures 1, 2, have been represented to showcase various types of DNA damaging alterations. White arrows represent the ruptured fragmented nucleus; white asterisks indicate micronuclei; yellow asterisks show herniated nuclei; and white triangles indicate blebbing with invagination. **(D)** Western blot images for different PTEN-mediated downstream stress-induced programmed cell death indication proteins, with their normalized value at different doses of mTOR inhibitor treatment against two different KSHV-infected cell lines. The reference gene expression of GAPDH has been used as loading control and utilized for all the other respective gene expression in each set.

Next, we were interested to reveal molecular direction of stress-responsive PTEN-mediated cell death events inside KSHV-infected cells. So, we have intended to analyze different modes of the PTEN-mediated stress-responsive process inside KSHV-infected cells in both PTEN-overexpressed and -silent conditions by using the serum stress-induced *in vitro* KSHV-infected cell line model. As PTEN is the major regulator of cell cycle, we first observed alteration of cell cycle phases in the stress-induced model. The cell cycle phase distribution pattern indicates PTEN-mediated restricted growth in the infected cells, following a synchronized cell death process with reduced proliferation in successive stages. KSHV-infected cells were found to be sensitive against PTEN overexpression and cell cycle arrest at the sub G0+G1 phase post serum stress recovery, whereas this process is hampered with PTEN silencing. On the other hand, during starvation recovery, normal KSHV-infected cells (without PTEN) can easily recover the stress, exerting normal cell cycle phase distribution. These results confirm the role of PTEN overexpression, culminating stress-induced senescence-mediated cell death (Figure 5A). Next, to access the role of PTEN over the mitochondrial stress-responsive cell death process in KSHV-infected cells, we performed JC-1 mitochondrial membrane potential assay in PTEN-overexpressed and -silent conditions against the stress-induced *in vitro* cell line model. We have observed that post stress exposure, mitochondrial stress is enhanced inside KSHV-infected cells, and KSHV-infected cells can successfully recover from this stress, whereas on PTEN overexpression/silencing, the enhanced mitochondrial stress cannot be manageable compared to control (Figure 5B).

Next, we checked the effect of mTOR inhibitor on KSHV cellular DNA. DAPI nuclear staining shows that the mTOR inhibitors are potential DNA damage inducers and were found to be effective against KSHV-infected cells, as has been analyzed (Figure 5C). The microscopic images clearly show that the mTOR inhibitor (at 20 nM dose) can effectively induce significant nuclear blebbing represented with white triangle compared to non-treated sets in the infected cells. Except this, invagination (indicated with yellow star) micronucleoli (represented in white star) and apoptotic bodies marked in white arrow has been observed with mTOR inhibitor treatment.

Now, after confirmation of the DNA damage, to further elucidate whether the inhibitor can induce any PTEN-mediated DNA damage response, we performed Western blot experiments to check the PTEN-mediated stress-sensitized cell death process. The death axis of PTEN-mediated stress-sensitized death has been analyzed by tracking the alteration of downstream molecular events. First, we have observed from our data that mTOR inhibitors can induce intercellular PTEN protein level during the cell death process along with subsequent reduction in its phosphorylated form KSHV-infected cells. This expression analysis indicates that mTOR inhibitors not only react depending on the presence of differential PTEN status inside the cell but can also modulate the PTEN protein status. Now, we intended to check the impact of the external DNA damage inducive agent (the mTOR inhibitor) on the PTEN-mediated stress-induced downstream cell death-identifying protein markers, where we found a significant dose-dependent, altered expression of ATM, p-ATM, chk1,chk2,p-chk2, cMYC, and p53 against different KSHV-infected cells (BC-3 and JSC-1) (Figure 5D). All these protein expressions post mTOR drug treatment clearly indicated that the mTOR inhibitor can exert PTEN-dependent ATM-mediated DNA damage response, resulting in the suppression of KSHV-infected cell growth identified by cell cycle checkpoint kinase activity (high level of both chk1 and chk2 level), which ultimately results in enhanced expression of p53, executing cell death. Moreover, we have also observed the gradual reduction of the cancer cell-identifying proto-oncogene cMYC with treatment, which also supports the fact of induction of apoptosis in the infected cells.

### Stress-responsive master regulator PTEN successfully regulates autophagy and apoptotic balance during stress management

**FIGURE 6:**
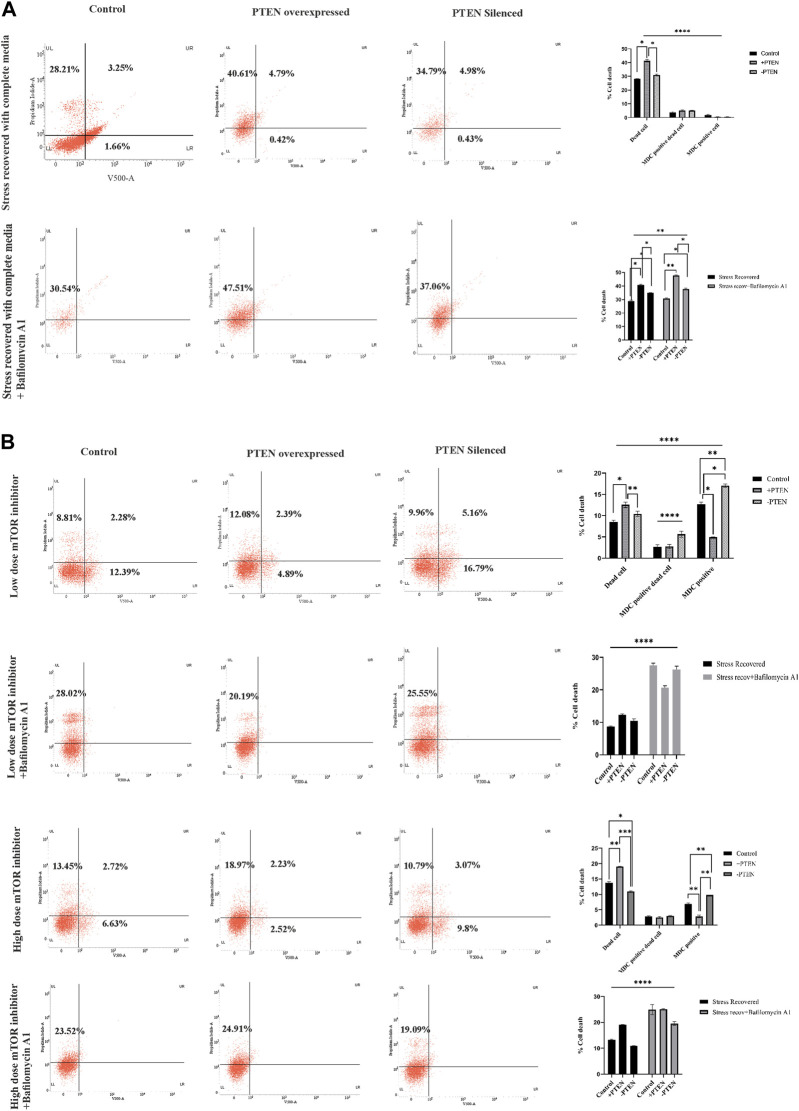
Status of autophagy with the concomitant cell death process during the PTEN-mediated cell death process in serum stress- and drug stress-induced KSHV-infected cell lines. V500 fluorescence channel represented in the x-axis of all the plots represents the autophagic intensity. **(A)** Dot plots showing autophagy vs. cell death in the serum stress model, with PTEN overexpression and silenced condition with or without bafilomycin A1. Comparative bar charts show the cell population percentage in each case. **(B)** Dot plots showing autophagy vs. cell death in both low- and high-dose mTOR inhibitor stress-induced model, with PTEN overexpression and silenced condition with or without bafilomycin A1. Comparative bar charts show the cell population percentage in each case.

It is important to elucidate the role of the stress-responsive PTEN-mediated death regulatory axis in connection with another stress balancing events like autophagy in KSHV-infected cells. So, in the next step, we have analyzed autophagy vs. cell death in the stress-induced *in vitro* KSHV cell line model in the presence or absence of PTEN. Our experiment shows that, in the stress-recovered cell, there is a clear autophagic upregulation and in PTEN-overexpressed and -silent sets, it is totally transformed to cell death without any autophagy positivity (Figure 6A, upper panel). To more specifically point out the autophagic event, we have performed a similar experiment with implication of autophagic inhibitor. The results clearly shows that autophagic impairment enhances the cell death process in all the experimental sets compared to control; moreover, the amount of dead cells are even higher in PTEN-positive and PTEN-silent sets. The PTEN-silent set shows lower amount of dead cells compared to the PTEN-overexpressed set. These results strongly establish involvement of PTEN in this process (Figure 6A, lower panel). The same process has been analyzed in the mTOR drug-induced cell line model to check whether the PTEN-mediated autophagic balance and cell death process works in same way as the stress-induced one. The results clearly demonstrate that at both low- and high-dose treatment of mTOR inhibitors, autophagy is transformed toward cell death in the PTEN transfected set, and the process is hampered by PTEN silencing. Moreover, autophagic suppression increases the cell death percentage in each case (Figure 6B).

## Discussion

It is important to explore the role of PTEN in different cancer models as PTEN is the master regulator of a varied group of cellular regulatory mechanisms, such as cell cycle regulation, apoptosis, and autophagy (Lee et al., 2018). Moreover, the endogenous PTEN status has been attributed as a major molecular identifying marker for cancer prognosis in response to different chemotherapeutic applications (Bazzichetto et al., 2019). In relation to this, it has been critically analyzed and observed in our study that endogenous PTEN genetic status and its functional properties attribute a major role during KSHV malignancy. Post-translational modulation of gene with its constitutive and functional expression during viral malignancy is a unique process to use the host cell mechanism for its benefit as a part of viral malignancy evolution (Li et al., 2021). PTEN acts as the upregulatory gene for the mTOR pathway. AKT-mTOR, the principal signaling cascade, regulated by PTEN, is phosphorylated at different molecular levels and is hyperactivated in KSHV-associated cancer due to the inactive PTEN’s efficacy (Bhatt and Damania, 2012). On the other hand, mTOR inhibitors are reported to exert promising effects by restricting cancer by their kinase activity targeting the downstream mTOR kinase molecules of PTEN in KSHV-infected cells (Mohanty et al., 2017). So, there is a fair chance that PTEN will also control the mTOR inhibitory drug regulation either in a positive or in a negative direction. Herpesviral cancer shows a unique mTOR-dependent transcriptional program executing rapamycin sensitivity (Chang and Ganem, 2013). Our study clearly shows that KSHV-infected cells harbor intact but phosphorylated PTEN that is functionally active and can create a reversible modulation in its downstream mTOR pathway, which makes these cells more responsive against the PTEN-mediated apoptotic process with mTOR inhibitors (Figures 1, 2). This indicates the fact that PEL cells do not develop any irreversible downstream mTOR modulation executing drug resistance where the PTEN gene acts as an essential determining factor for therapeutic efficacy of the mTOR inhibitors. Though PTEN has been reported as a wild-type gene, its quantitative expression variation with control cells has not been reveled yet in KSHV-infected cells (Roy and Dittmer, 2011). Our study shows that the PTEN gene is expressed much higher level in KSHV-infected cells compared to healthy PBMC, but the KSHV transcriptional program executes post-translational modification of the gene and vFLIP stabilizes the function of PTEN; thus, the active form of PTEN is suppressed and inactivated during malignancy (Figure 3D). Moreover, in our study, we utilized serum stress to track the functional direction of PTEN, where PTEN has been found as a stress-responsive cellular partner and stress manager (Figure 3A), controlling the oncogenic stress response and switch toward stress-mediated cell death in response to ROS by utilizing the stress-induced *in vitro* KSHV-infected cell line model (Figures 3B, C). Our results clearly show that post-translationally modulated, hyperphosphorylated PTEN results in hyperactivation of its downstream mTOR signaling pathway and makes these cells an ideal therapeutic target by mTOR inhibitors, whose sensitivity is determined by intercellular PTEN in KSHV-infected cells (Figure 4). PTEN overexpression can successfully divert the drug-induced stress toward apoptosis both at low and high doses.

On this note, it needs to be mentioned that Burkitt’s lymphoma cells are sensitive against PTEN overexpression by regulating the downstream PI3K-AKT pathway controlling cell cycle and apoptosis regulation (Li et al., 2020). Moreover, another member of the herpesvirus family, close to KSHV, EBV-associated nasopharyngeal carcinoma, has been reported to exert PTEN-dependent viral regulation enhancing metastasis. Overexpression of PTEN has shown a promising result controlling this metastatic event (Cai et al., 2015). It indicates that whether it is infectious or non-infectious B-cell lymphoma, they show PTEN-induced death sensitivity. In relation to this, our comparative analysis of functional importance of different lines of PTEN modulation in B-cell lymphoma justifies the significance of the presence of endogenous PTEN in exerting enhanced drug sensitivity compared to the PTEN-deleted condition (Figure 2B). In this regard, we have observed that the overexpression of PTEN showed a significant level of enhanced stress-mediated cell death induction, whereas silencing PTEN does not seem to significantly alternate the process, which indicates the fact that although PTEN supports the cell death process, it is not solely dependent on PTEN (Figure 3). Hence, the role of PTEN as a drug sensitivity or resistance marker needs further clarification with its long-term effect.

Proper nuclear–cytoplasmic distribution of PTEN is important for the execution of PTEN activity of initiation programmed cell death process (Chung and Eng, 2005). The PEL xenograft model, KSHV tumor samples, and KSHV-infected cell lines have been reported to possess PTEN and p-PTEN uniform localization in both the nucleus and cytoplasm (Roy and Dittmer, 2011). The functional importance of this intact and well-distributed tumor suppressor has been analyzed with application of conditional media in our study. Here, we found that serum stress acts as the conditional factor for PTEN expression and its distribution between the nucleus and cytoplasm in normal cells and KSHV-infected cells, whereas KSHV FLIP protein was found to be cytoplasmically co-localized with PTEN. Previously, it has been reported that cFLIP regulates the stress management system in normal cells and viral mimic proteins, and vFLIP is reported to act as a stress balancer agent by managing the stress-induced signaling (Lee et al., 2009; Mora-Molina et al., 2022). In relation to this, our findings of vFLIP–PTEN colocalization might be an indication toward its role of stabilizing the stress-responsive PTEN’s distribution and its activity. This finding can be supplemented by a previous report of PTEN-mediated FLIP protein stabilization during cancer (Panner et al., 2009). Our study gives a therapeutic probability as we show that this stabilization process (PTEN–vFLIP) can be therapeutically hampered by the application of mTOR inhibitors (Figure 2).

In the next part of our study, we have proceeded to check the functionality of PTEN as a stress-sensitized cell death induced from a different angle in terms its therapeutic possibilities with the application of therapeutic stress. For this, we have further analyzed the PTEN-mediated regulation of different stress-responsive cellular functioning, like cell cycle regulation and mitochondrial stress response, which strongly proves that PTEN can initiate a stress-sensitized synchronized cell death program inside KSHV-infected cells post mTOR inhibitor treatment. On this note, it needs to be mentioned that dysfunctional mitochondria exerts cellular transformation in different cancers. Proapoptotic PTEN has been reported to act as a quality controller of the ROS-induced mitochondria-generated stress management process through mitophagy and induction of mitochondrial apoptosis (Zhu et al., 2006; Gough, 2014; Li et al., 2018). Our result clearly shows that PTEN can successfully induce the mitochondria-mediated cell death process in KSHV-infected cells. These results collectively demonstrate that KSHV-infected cells exert differential expression of PTEN during stress and normal conditions as a part of the oncogenic stress management system. Moreover, KSHV cells follow the same route to exert stress-induced cell death by enhancing cellular senescence and disrupting mitochondrial membrane potential.

Oxidative stress-induced PTEN-mediated cell death has previously been reported to work in the ATM-p53 axis in cancer cells (Chang et al., 2008; Chen et al., 2015). Our molecular analysis of the mTOR-mediated death program clearly revealed a sequential regulatory axis of PTEN-mediated cell death induction in KSHV-infected cells, where mTOR inhibitors function as important kinase inhibitors and serve as a potential inhibitor for PTEN phosphorylation and enhance the role of active PTEN, which induces oncogenic stress-sensitized ATM-dependent DNA damage-induced cellular senescence with the involvement of checkpoint kinase regulation. This event is sequentially headed toward p53-mediated mitochondrial apoptosis (Figure 5). Hence, these results showed an indication that mTOR inhibitors can be an efficient therapeutic agent to target PTEN modulation in KSHV-infected cells.

In the last part of this study, we determined the functionality of PTEN in relation to autophagic stress regulation in KSHV cells. KSHV cells show a varied level of autophagy throughout its life cycle from infection, establishment, maintenance, and reactivation. FLIP-mediated autophagy subversion for latency maintenance is an important event inside KSHV-infected cells for stabilizing them from the stress-sensitized cell death process (Lee et al., 2009). mTOR is the major cellular regulator for autophagy which is upregulated during KSHV malignancy, exerting autophagic suppression, which is reported to create metabolically weak cancer cells, making them sensitized against targeted therapy (Gremke et al., 2020). Autophagy which has been well analyzed as the cellular stress controller has been reported to be controlled by tumor suppressor PTEN as a therapeutic target during cancer (De Amicis et al., 2014; Aquila et al., 2020). Our study proves that PTEN is the regulator for mTOR and mTOR drug sensitivity, so we further intersected the status of autophagy in the PTEN-mediated mTOR-dependent cell death process in KSHV-infected cells. In our previous study, we have analyzed that oncogenic stress response is an ongoing dynamic process to control any kind of stress induction in a KSHV-infected cancer microenvironment. In a drug-induced stressed environment, there is competition between oncogenic stress response and cellular stress response. We clearly investigated that this PTEN-mediated stress response is totally autophagy dependent. PTEN has been found as the molecular master switch node of autophagy apoptosis (Figure 6). The stress regulatory process autophagy plays the key role as the determining factor between these two stress regulatory responses. Autophagic response decides whether a cancerous cell will undergo cell death by overcoming the oncogenic stress response or continue to proliferate. Autophagy may help the cell to overcome stress induction, thus helping the process of adaptation either by cell death with reactivation or drug-insensitive cellular proliferation, ultimately making the cell resistant to chemotherapy (Das et al., 2022). Autophagy is mTOR regulated and can act as stress-responsive signaling in overcoming any kind of stress exposure. Our previous report showed an upregulated autophagy post everolimus treatment in KSHV-infected cells at initial doses which fails to protect the apoptotic induction by the drug (Mohanty et al., 2017). Moreover, phosphorylated PTEN translocates toward the nucleus by oxidative stress induction and induces autophagy and p53-dependent apoptosis, as previously reported (Chen et al., 2015). So, we checked the status of PTEN-regulated autophagy in KSHV-infected cells. Our results showed that during stress induction, PTEN translocation and autophagic upregulation happen, which is for managing the stress level, and the presence of *wt* PTEN is important for a stress-exposed KSHV cell to manage the extra stress burden through autophagy. This clarifies the importance of autophagic upregulation at initial dose and this mechanism, in turn, makes the infected cells sensitive against mTOR inhibitors. PTEN overexpression actually sensitizes this stress induction and induces DNA damage and p53-mediated apoptosis, which is autophagy dependent. That is why, in PTEN mutant B-cell lymphoma, post mTOR inhibitor treatment, a high level of autophagy induction is observed compared to *wt* PTEN-harboring KSHV cells. In the case of PTEN mutant, the cell’s autophagic upregulation is protecting the cells against mTOR-mediated cell death, whereas in the case of KSHV-infected cells, the *wt* PTEN controls the autophagy and directs it toward successful apoptosis. High-dose mTOR inhibitor treatment also follows the same route. So, it can be concluded from the aforestated fact that PTEN modulates mTOR and mTOR modulates autophagy in a subvert condition during KSHV malignancy by hijacking the healthy cell’s stress regulatory route, which opens a therapeutic window for “targeted therapy” by making the cell sensitize against stress-sensitized PTEN-mediated autophagic-induced mTOR-regulated cell death.

**FIGURE 7:**
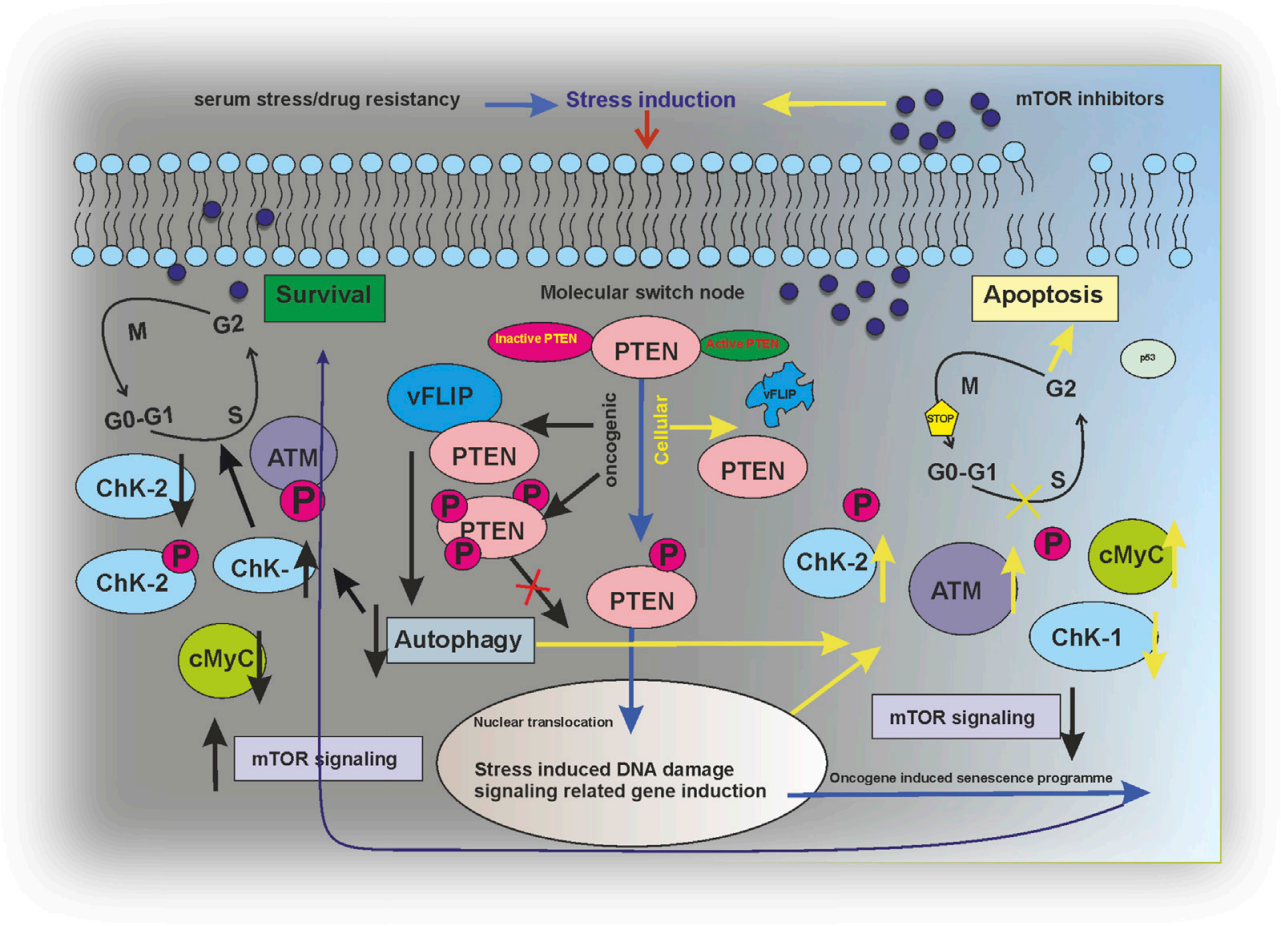
Regulatory modeling of PTEN acting as a molecular switch node between survival and apoptosis in an mTOR inhibitor-induced environment.

The current study has successfully figured a PTEN-mediated mTOR kinase inhibitory drug-sensitive death axis can be activated in KSHV-infected cells (Figure 7). Moreover, our overall study shows the impact of post-translationally modulated PTEN in KSHV-infected cells, which, in turn, makes these cells the target point for mTOR kinase inhibitors and strongly indicates the fact that these group of drugs are the best fit against this type of virus-infected cancer cells with PTM (post translational modification). As PTM seems as the evolving diagnostic and therapeutic stand point in cancer studies (Zhang and Han, 2020; Meng et al., 2021), this study shows the path to think whether these virus-associated cancer types having PTM of PTEN develop mTOR inhibitor drug resistance by mTOR kinase modulation compared to other cancer types with deleted PTEN, which will reveal the therapeutic target point evolution for new-generation mTOR inhibitors to work better in such a cancer setup.

The current study is limited only to understanding the functional importance of PTEN in KSHV-infected cells with respect to its therapeutic standpoints. The impact of the interaction of viral gene vFLIP with PTEN and its importance need further investigation in terms of host–viral interaction. Moreover, as this study shows an indication of the involvement of PTEN in determining mTOR inhibitory drug sensitivity, the same functionally needs detailed understanding and assessment in an mTOR inhibitor therapy-resistant model in the presence or absence of PTEN.

## Data Availability

The raw data supporting the conclusion of this article will be made available by the authors, without undue reservation.
