# Supplementary figures and images for: Endogenous PTEN acts as the key determinant for mTOR inhibitor sensitivity by inducing the stress-sensitized PTEN-mediated death axis in KSHV-associated malignant cells
Source: Front Mol Biosci. 2023 Aug 2;10:1062462. doi: 10.3389/fmolb.2023.1062462 (PMC10433768; doi:10.3389/fmolb.2023.1062462)

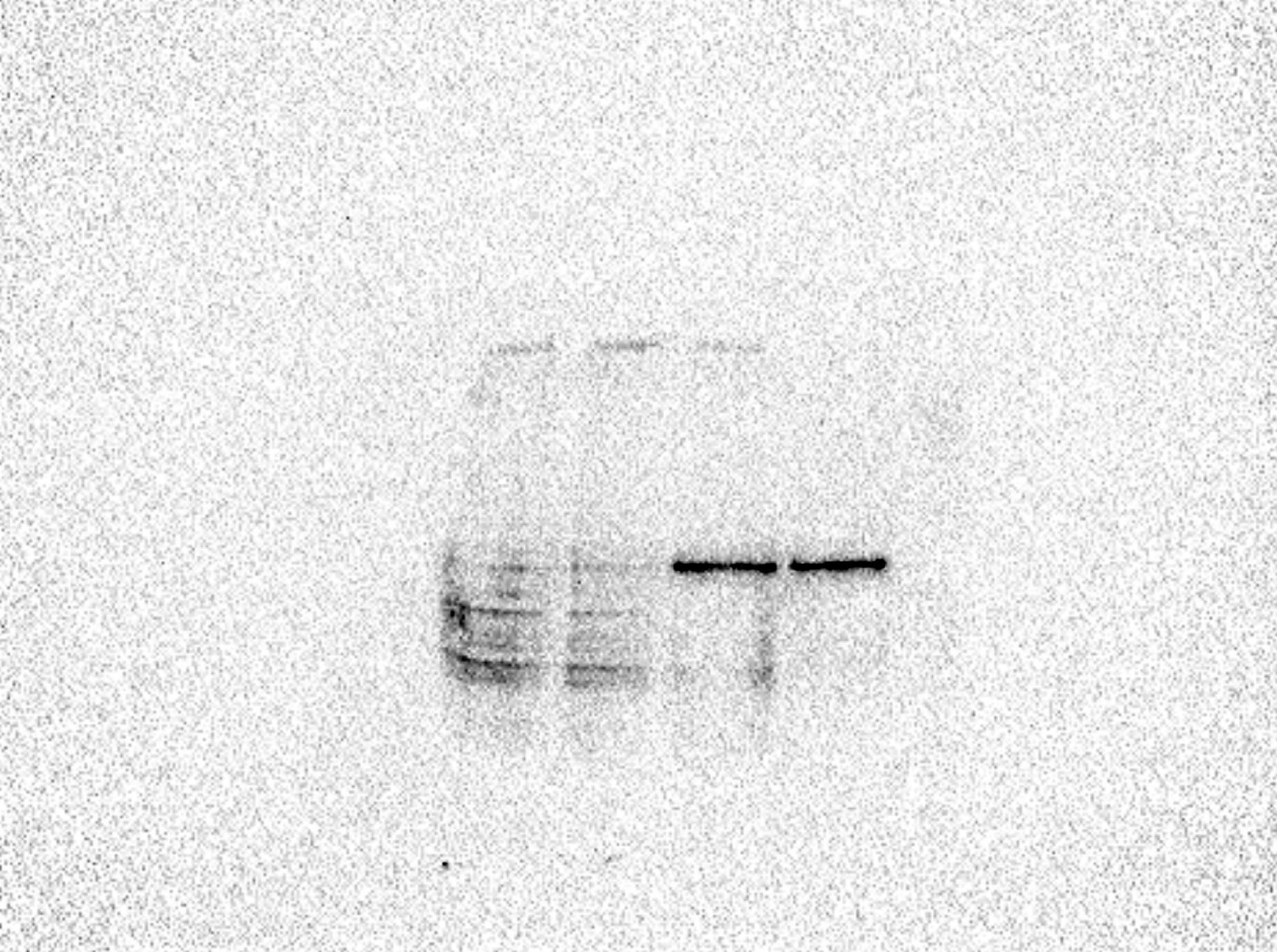

Supplement: Supplementary file 1 [file DataSheet1.zip › BC-3/ATM.tif]

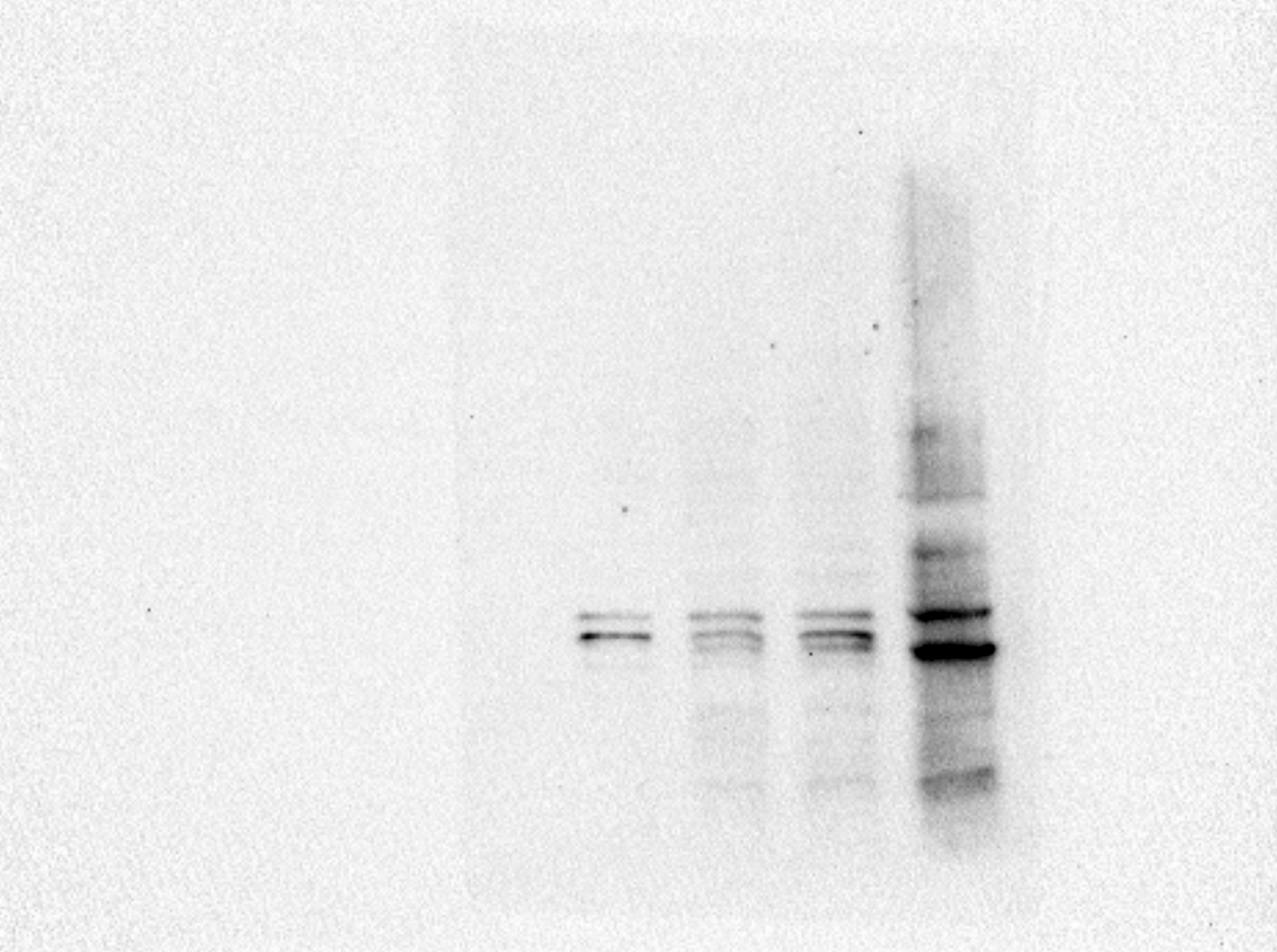

Supplement: Supplementary file 1 [file DataSheet1.zip › BC-3/ABC 2018-01-26 20hr 13min_Exposure_100.0sec cmyc.tif]

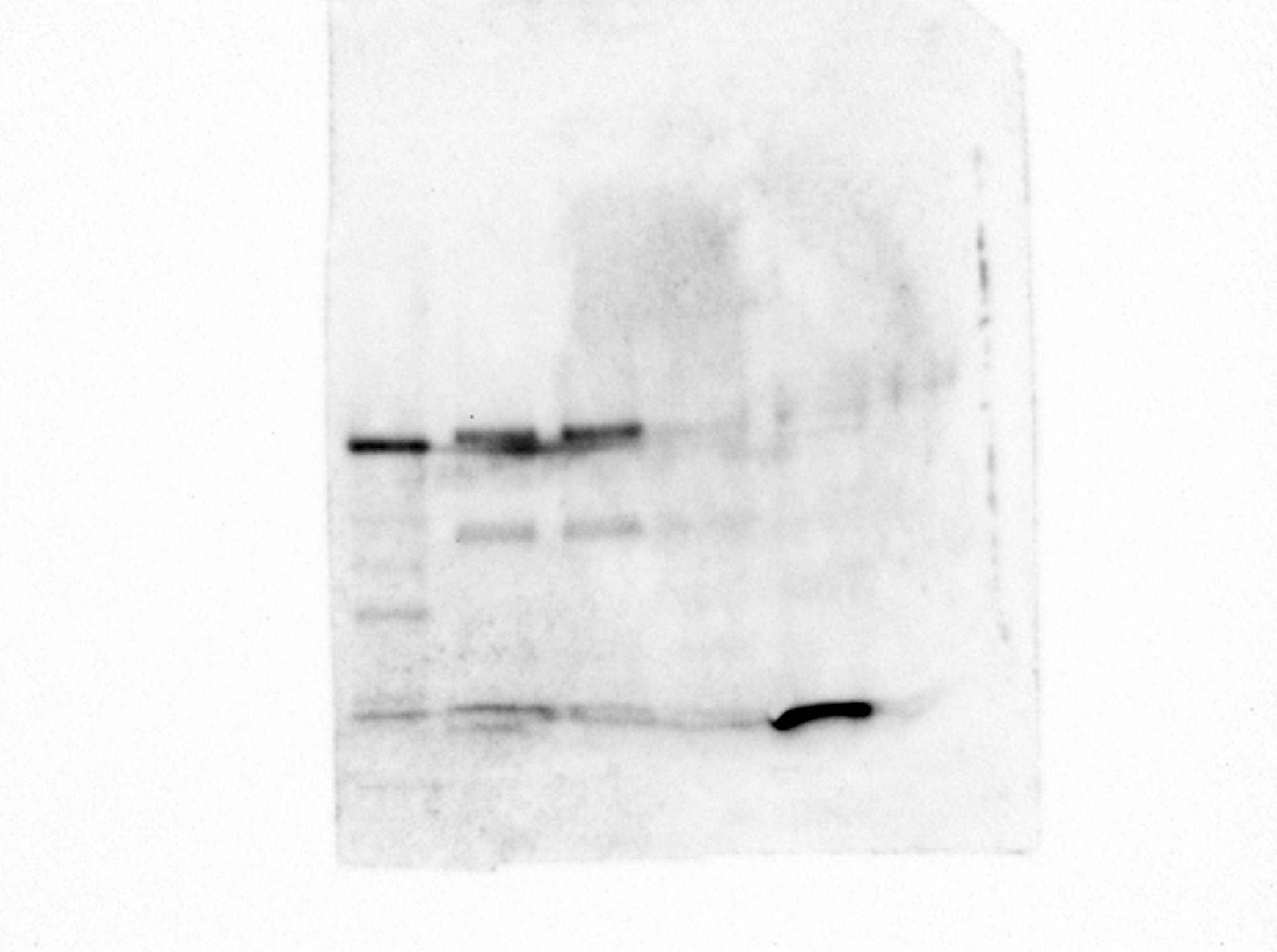

Supplement: Supplementary file 1 [file DataSheet1.zip › BC-3/pATM.tif]

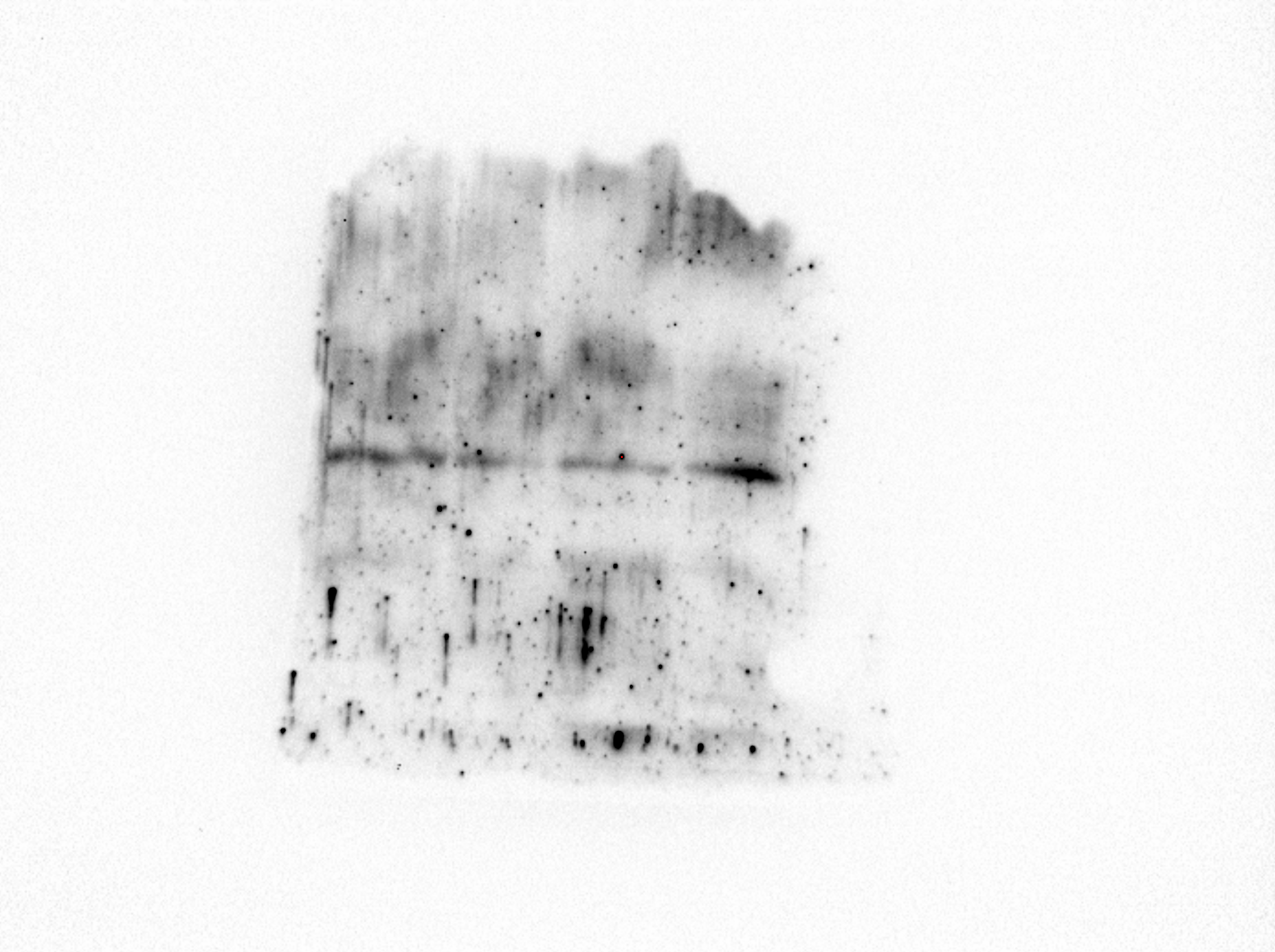

Supplement: Supplementary file 1 [file DataSheet1.zip › BC-3/PTEN.tif]

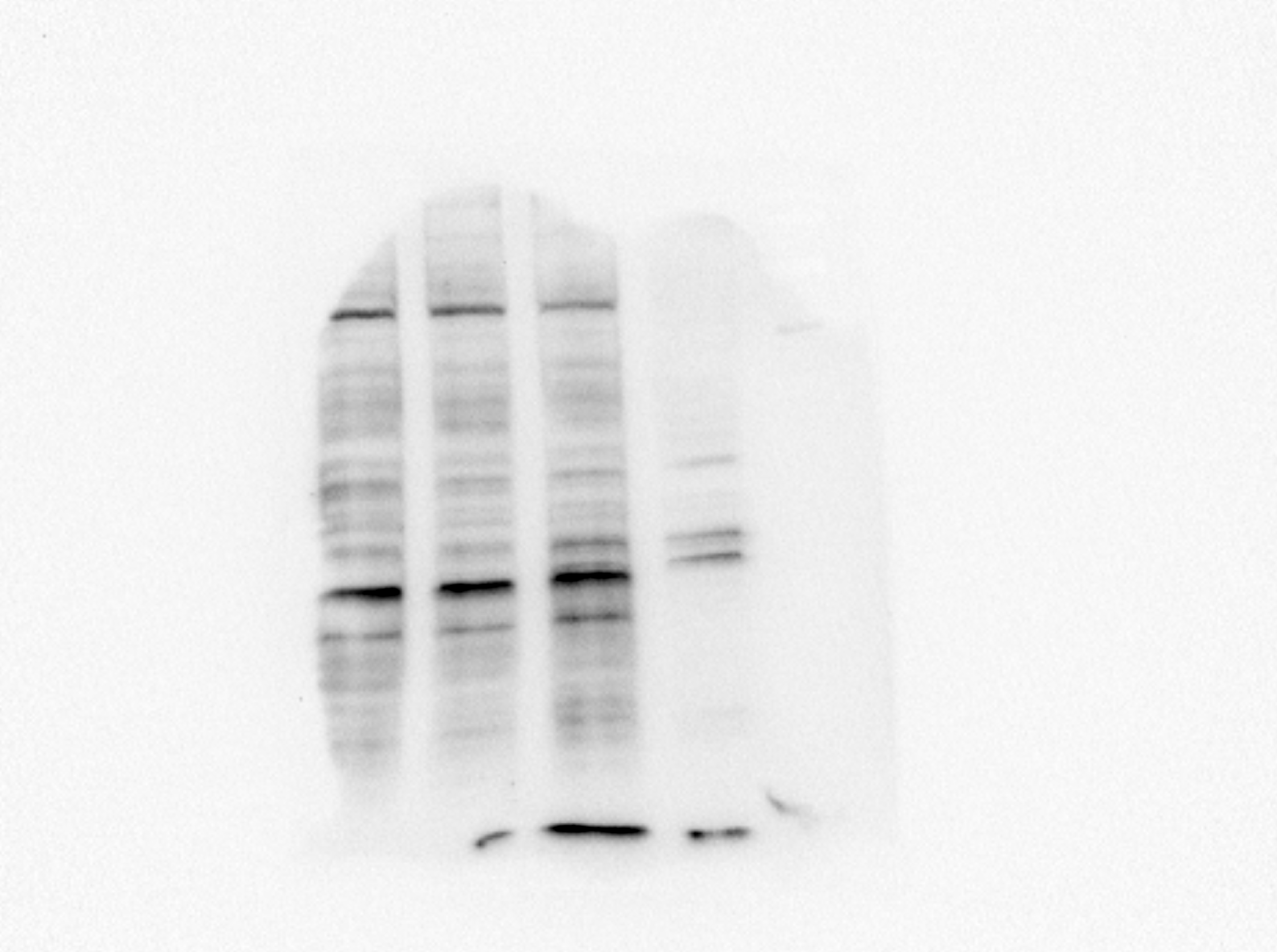

Supplement: Supplementary file 1 [file DataSheet1.zip › BC-3/CHK-2.tif]

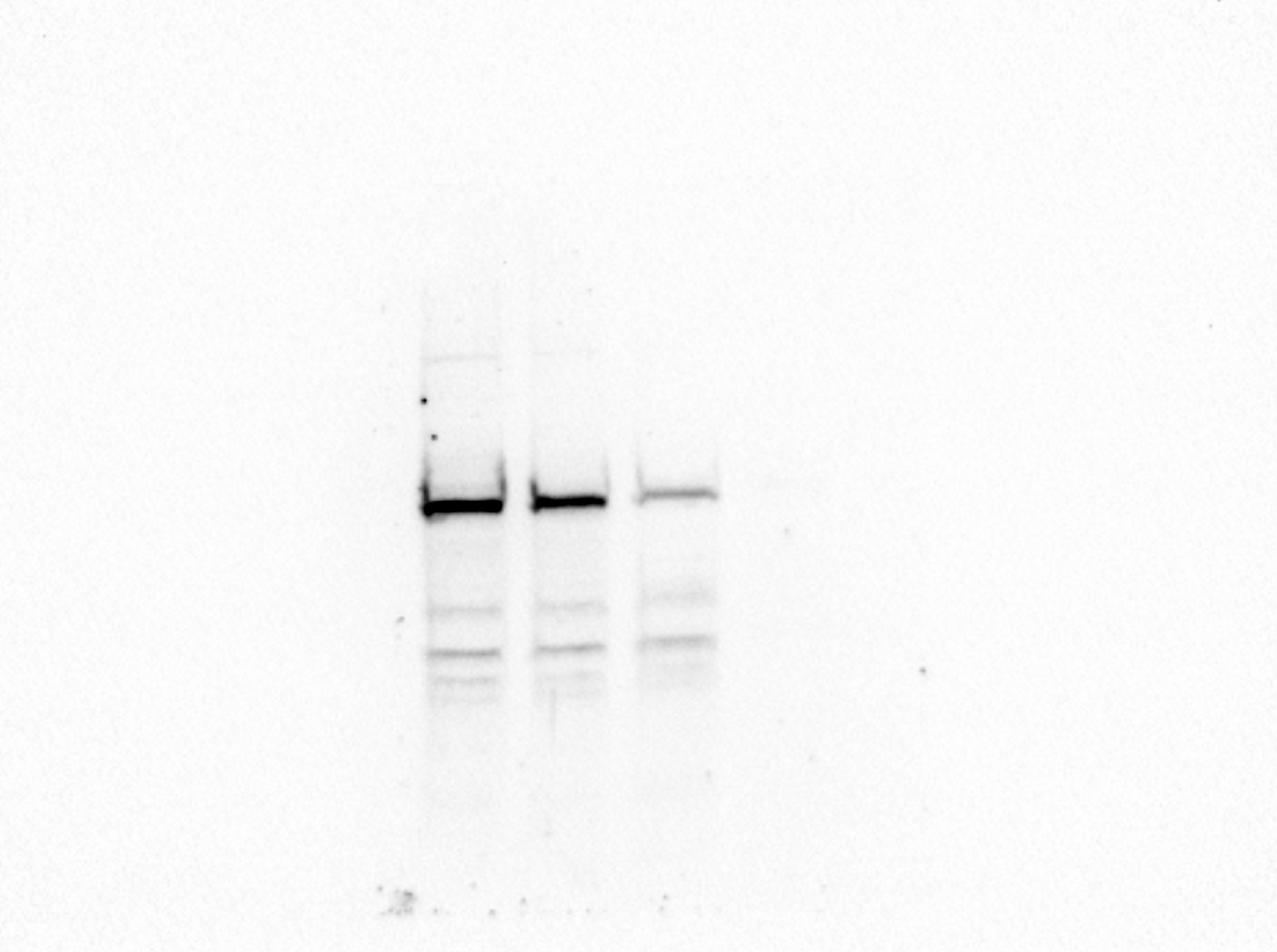

Supplement: Supplementary file 1 [file DataSheet1.zip › BC-3/Chk-1.tif]

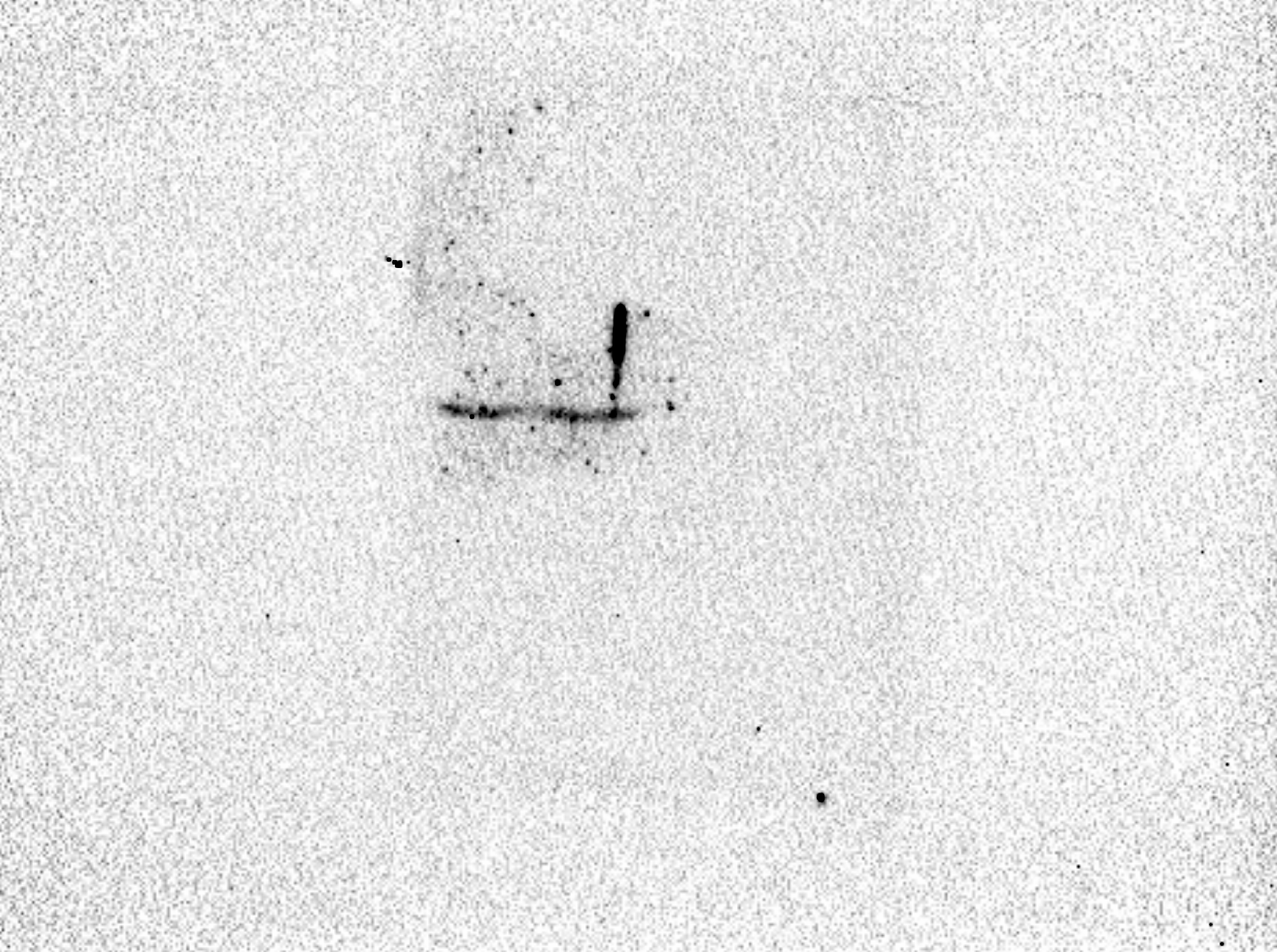

Supplement: Supplementary file 1 [file DataSheet1.zip › BC-3/P chk2.tif]

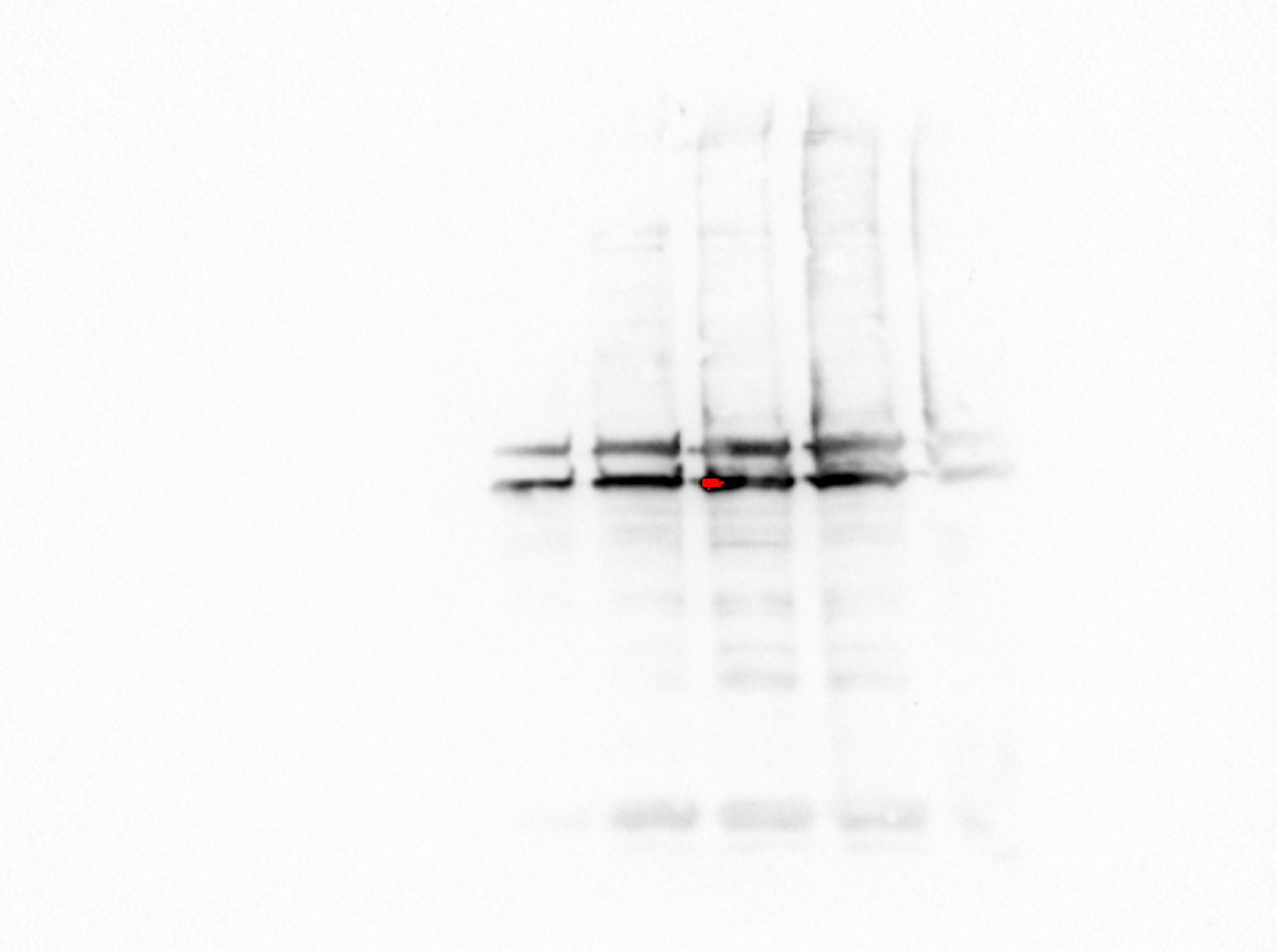

Supplement: Supplementary file 1 [file DataSheet1.zip › BC-3/GAPDH.tif]

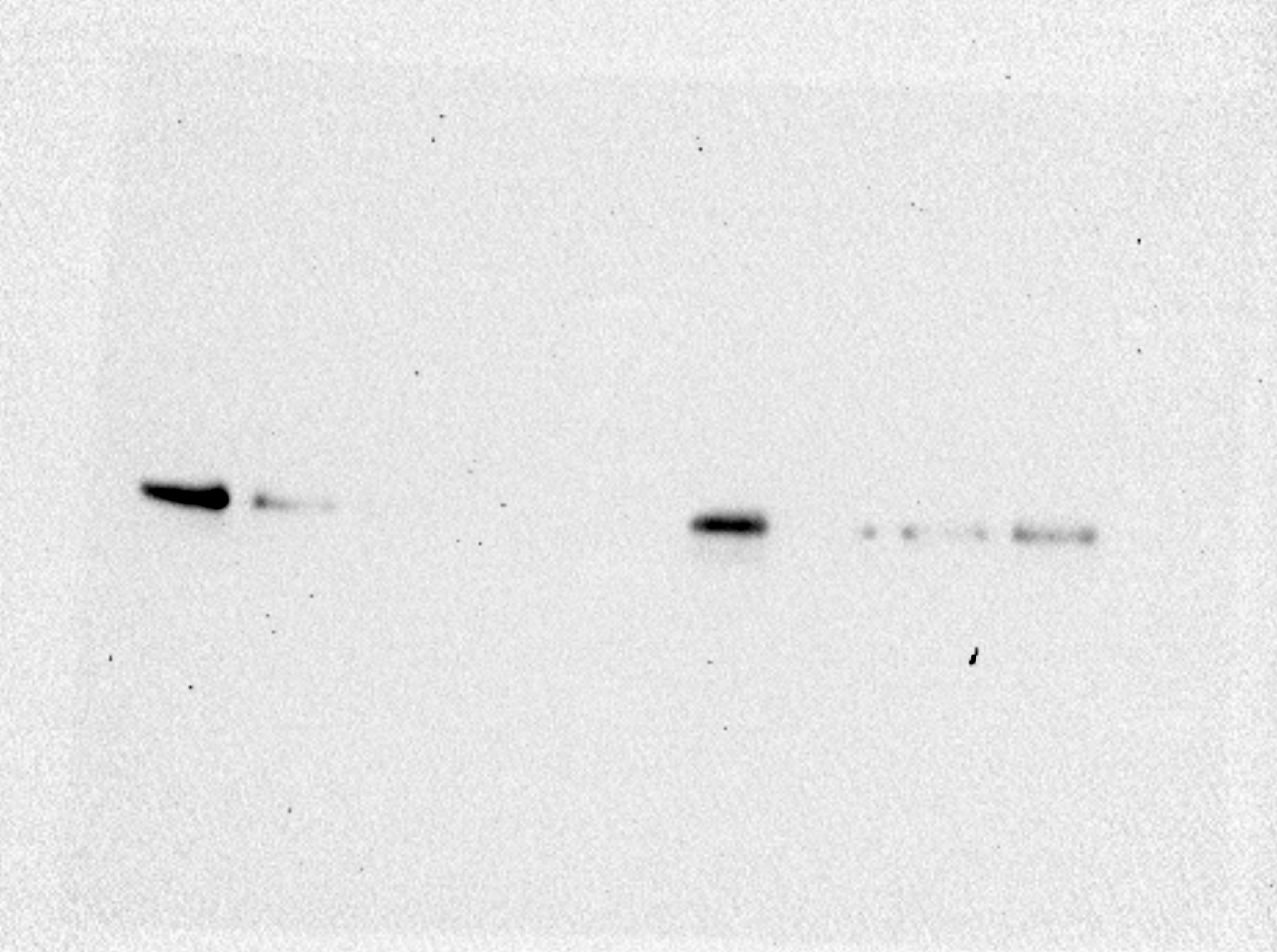

Supplement: Supplementary file 1 [file DataSheet1.zip › BC-3/p-PTEN.tif]

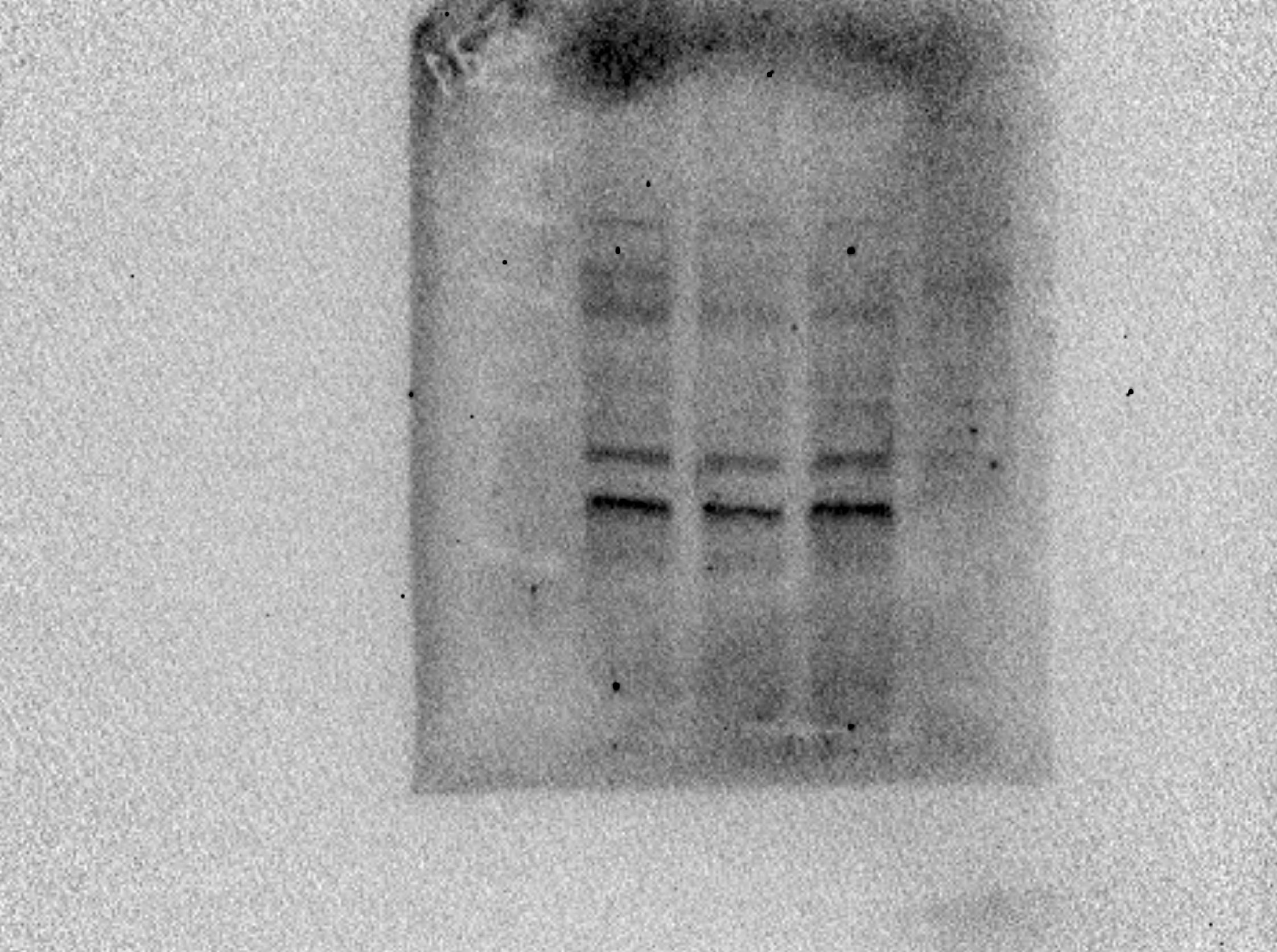

Supplement: Supplementary file 1 [file DataSheet1.zip › BC-3/p53.tif]

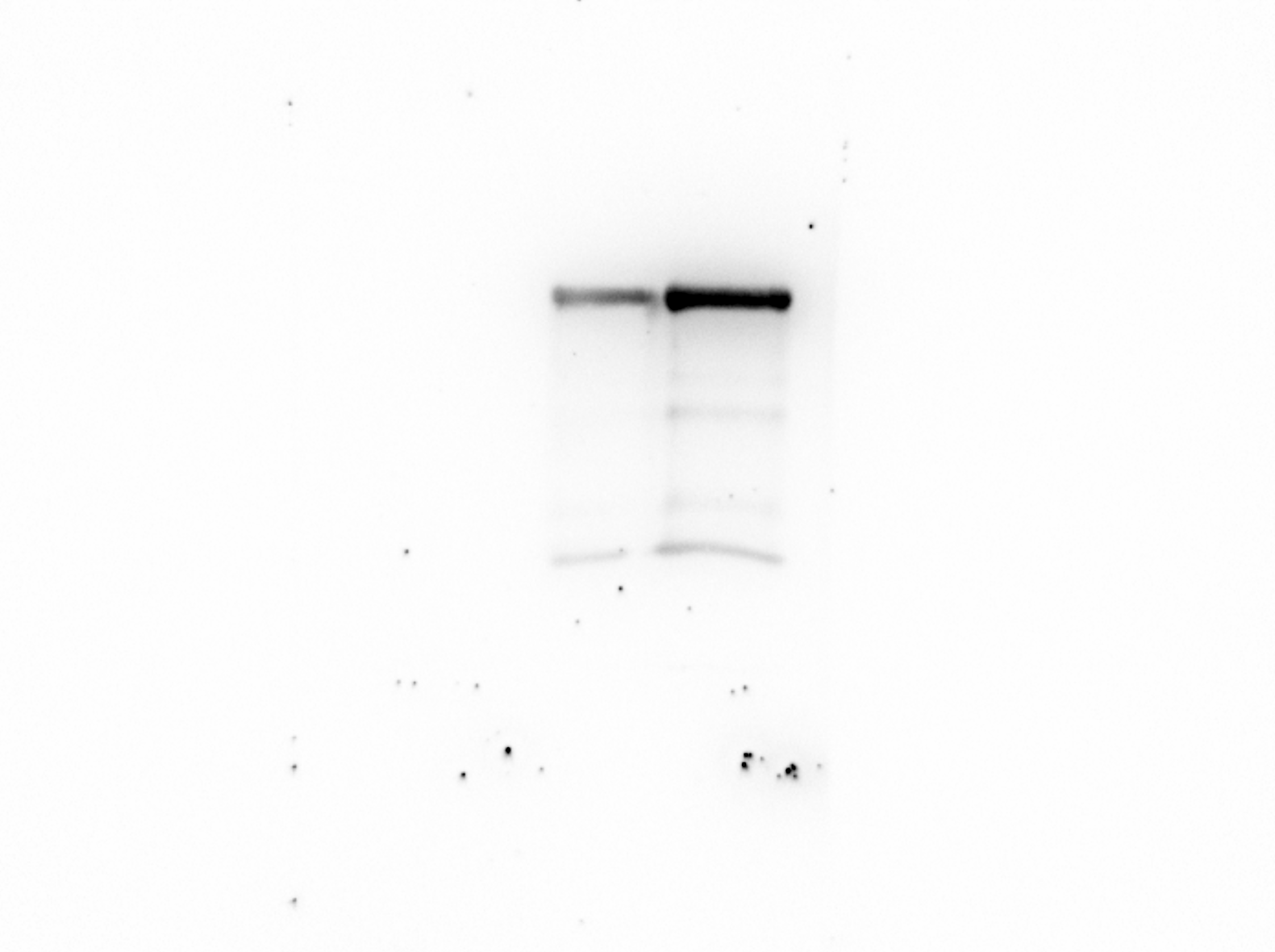

Supplement: Supplementary file 2 [file DataSheet2.zip › JSC-1/ATM.tif]

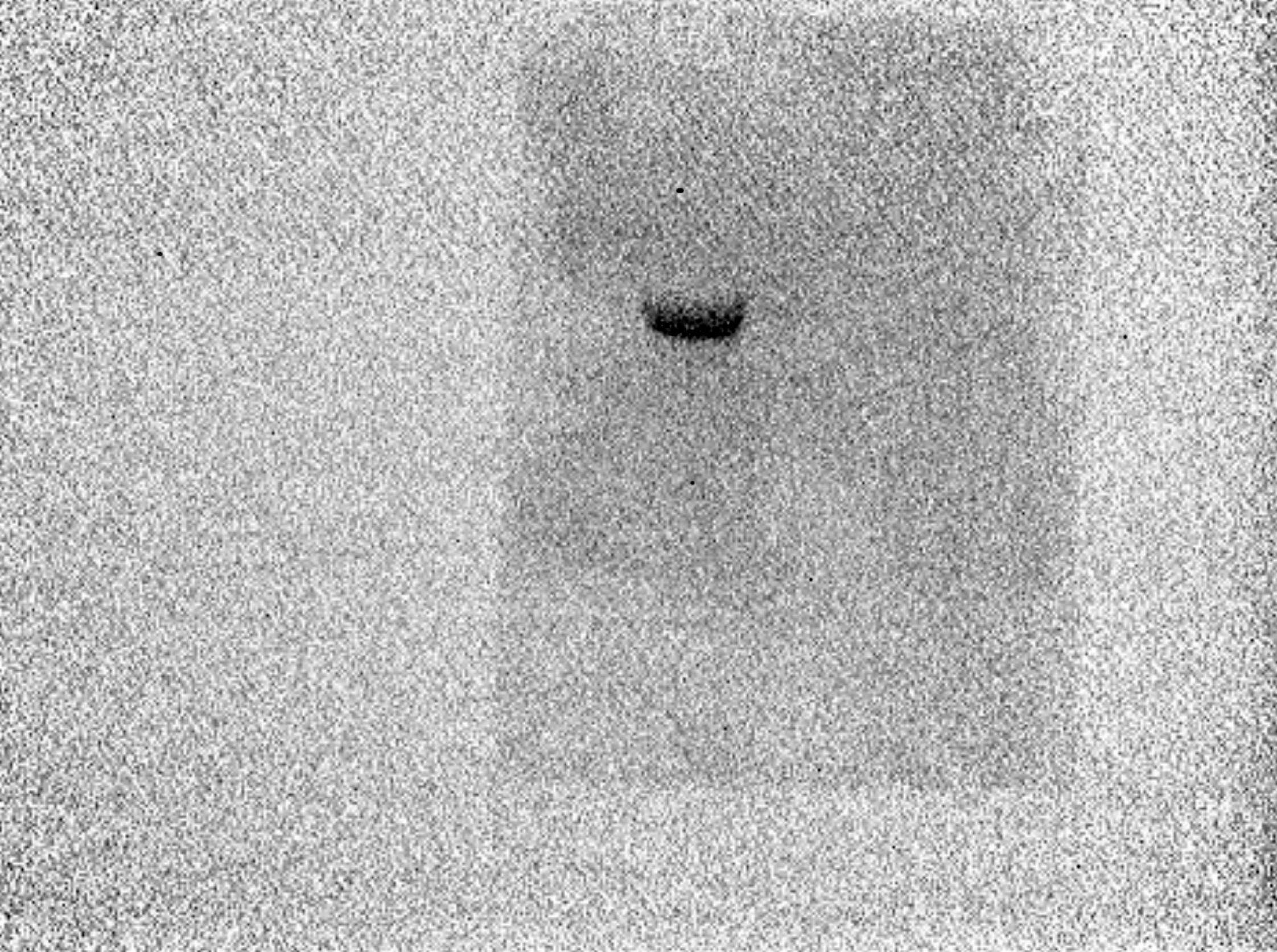

Supplement: Supplementary file 2 [file DataSheet2.zip › JSC-1/p PTEN.tif]

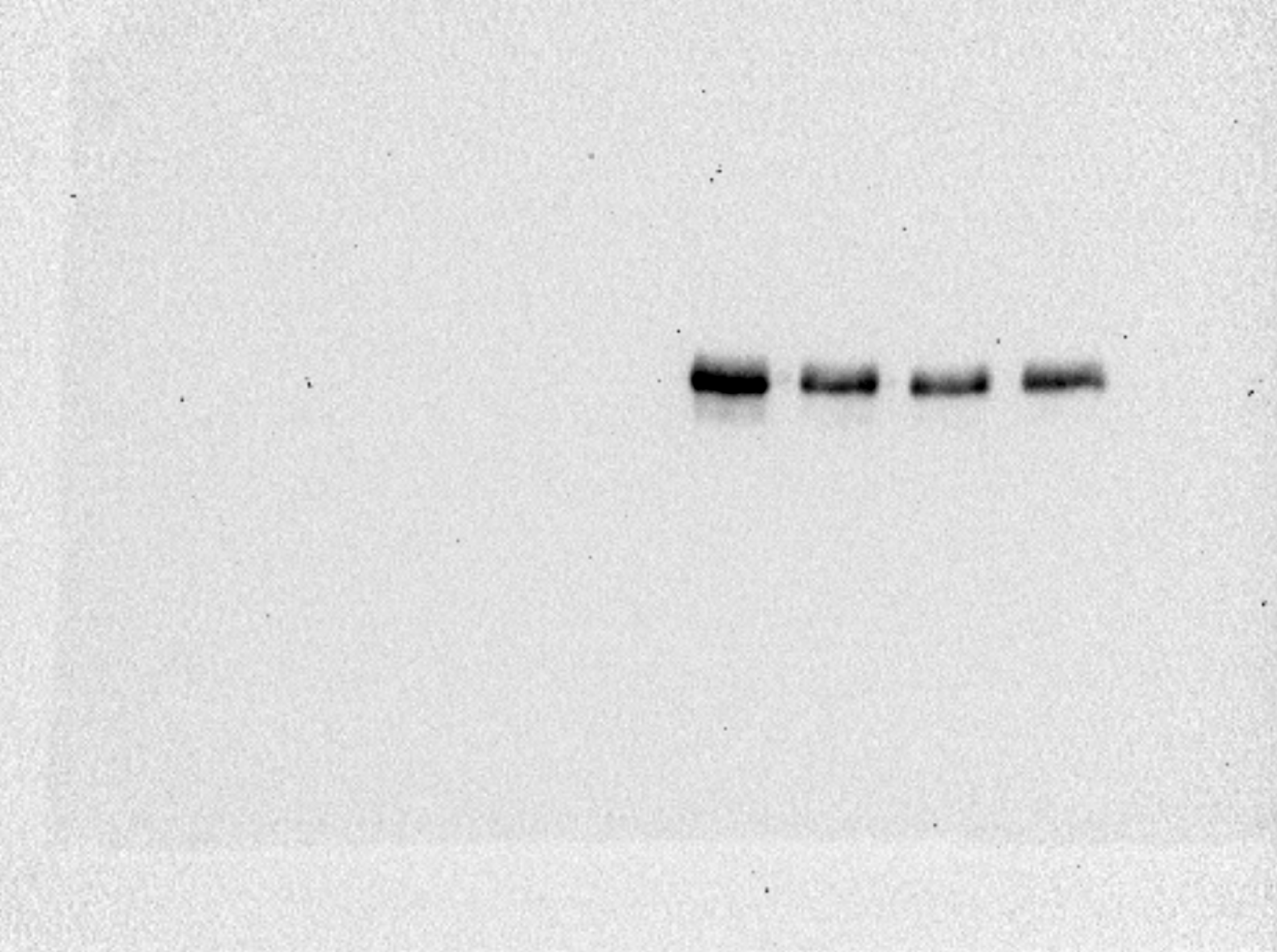

Supplement: Supplementary file 2 [file DataSheet2.zip › JSC-1/PTEN.tif]

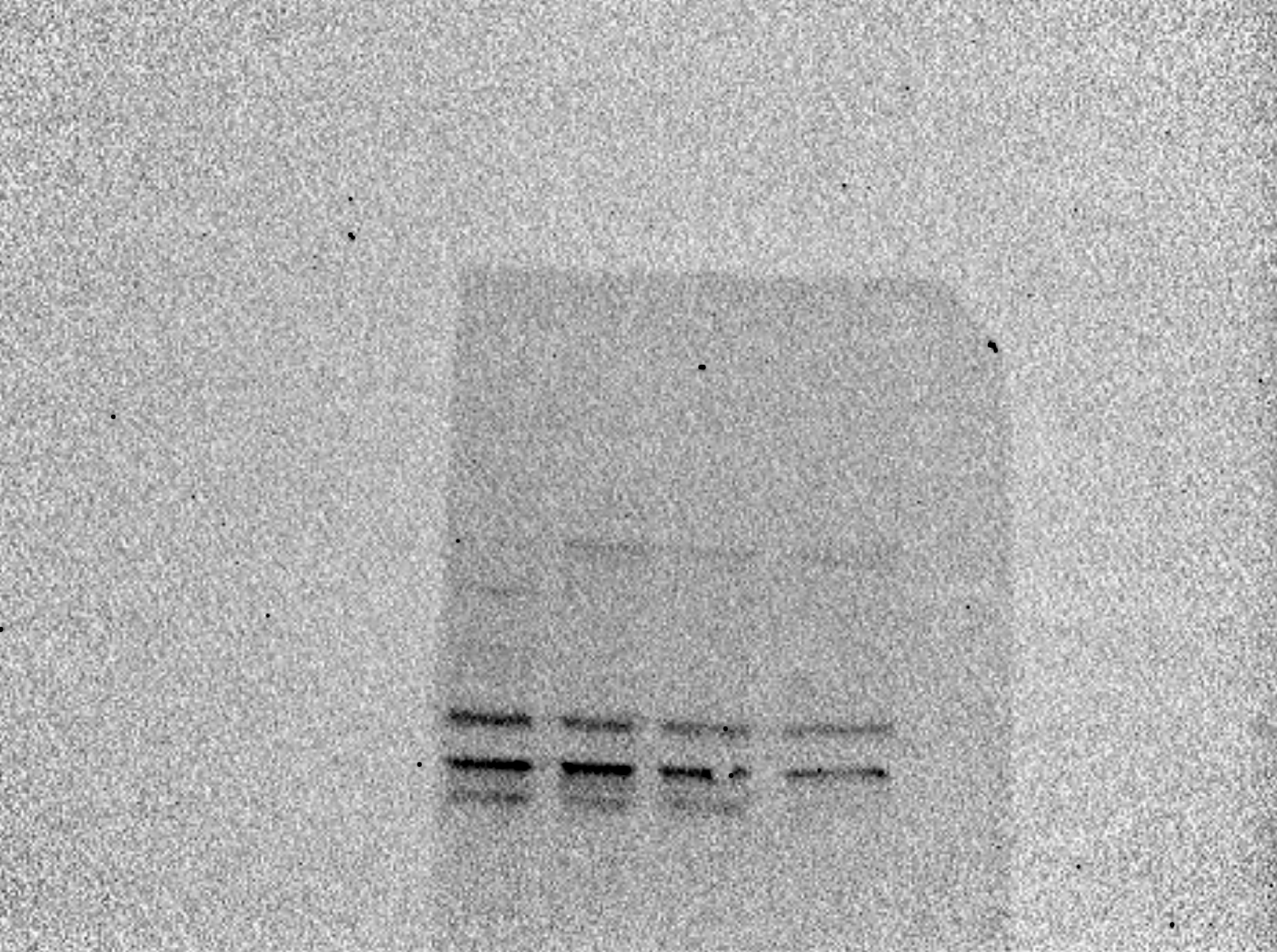

Supplement: Supplementary file 2 [file DataSheet2.zip › JSC-1/CHK-2.tif]

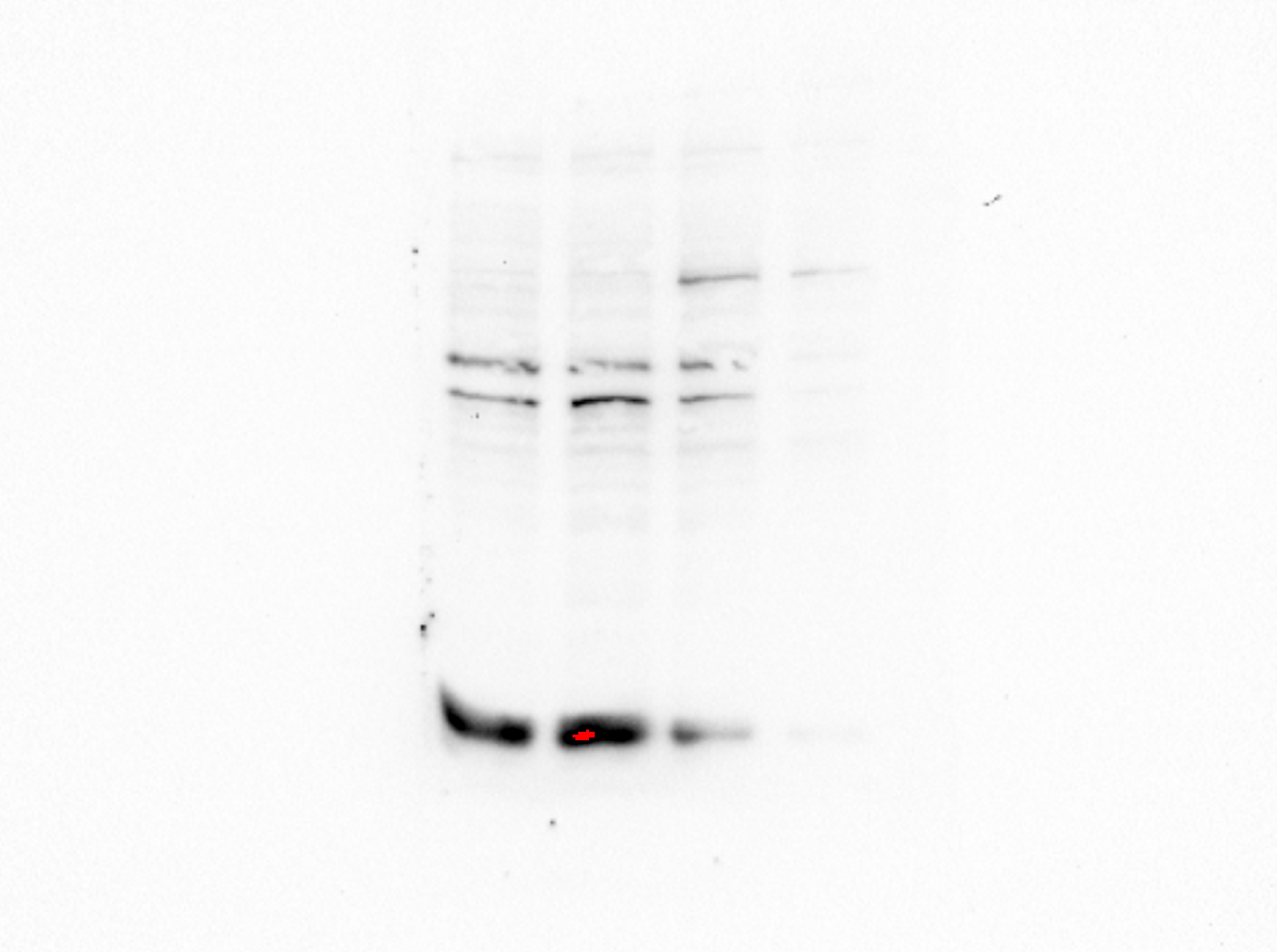

Supplement: Supplementary file 2 [file DataSheet2.zip › JSC-1/CHK-1.tif]

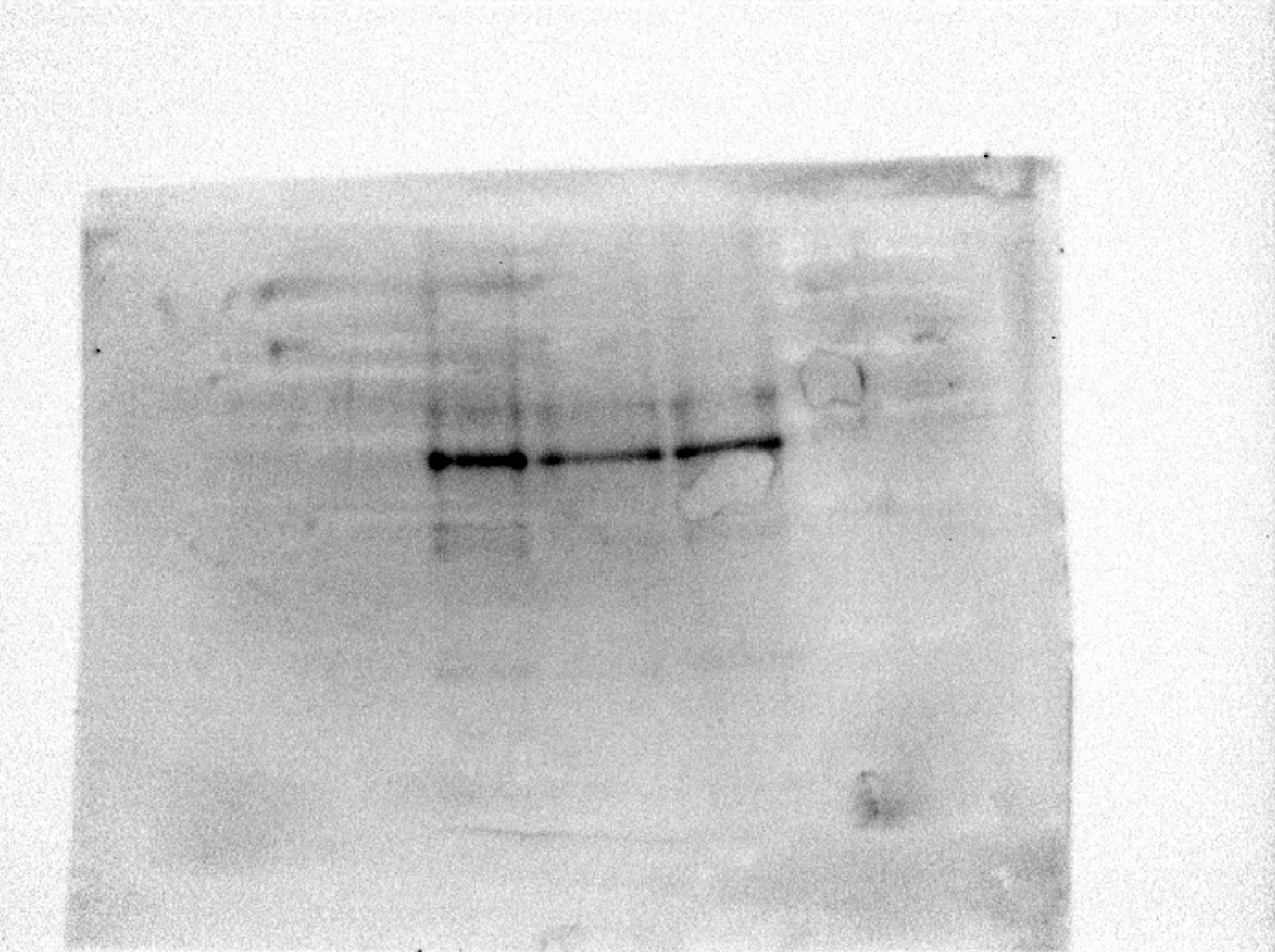

Supplement: Supplementary file 2 [file DataSheet2.zip › JSC-1/p53.jpg]

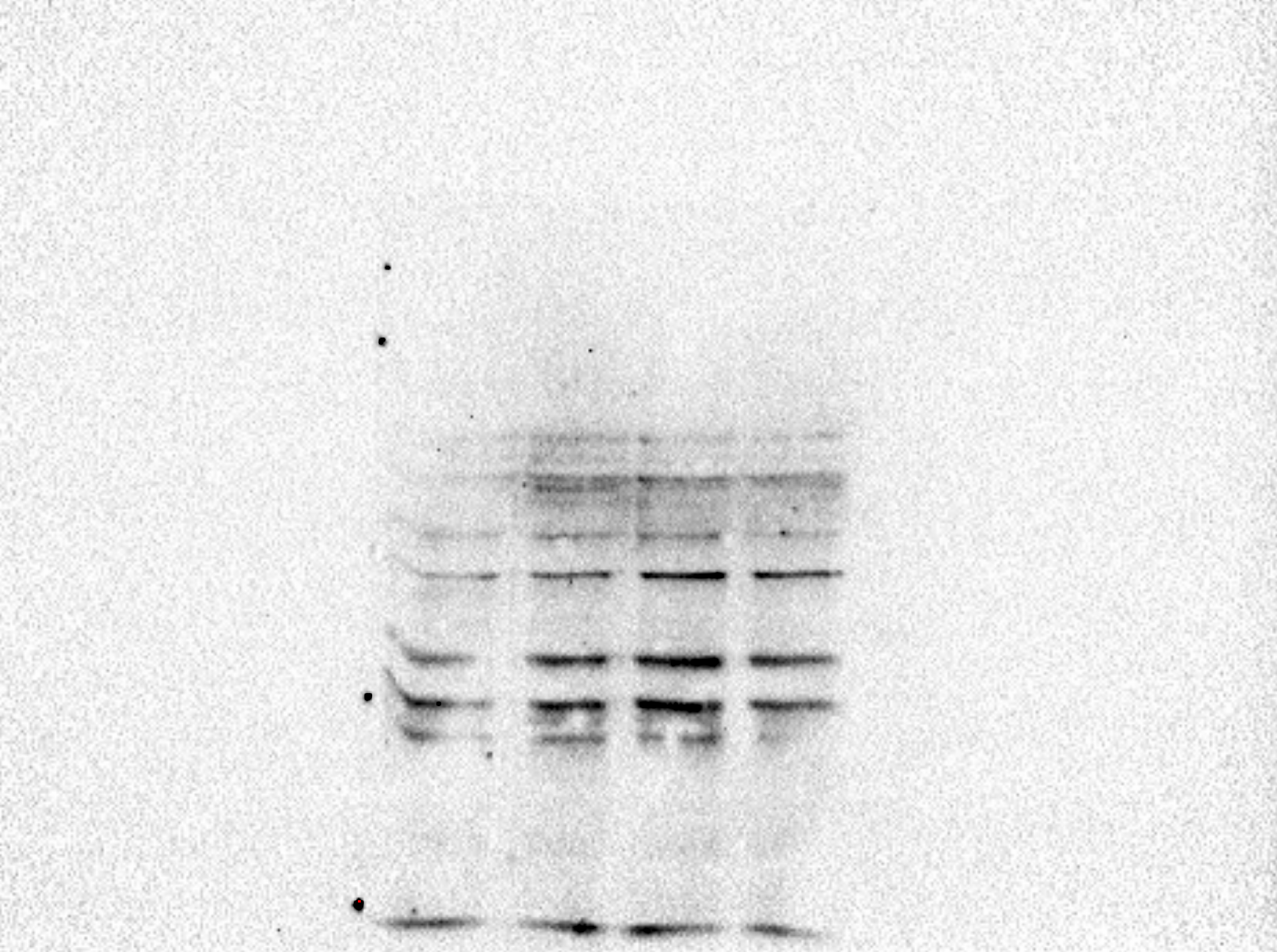

Supplement: Supplementary file 2 [file DataSheet2.zip › JSC-1/P-ATM.tif]

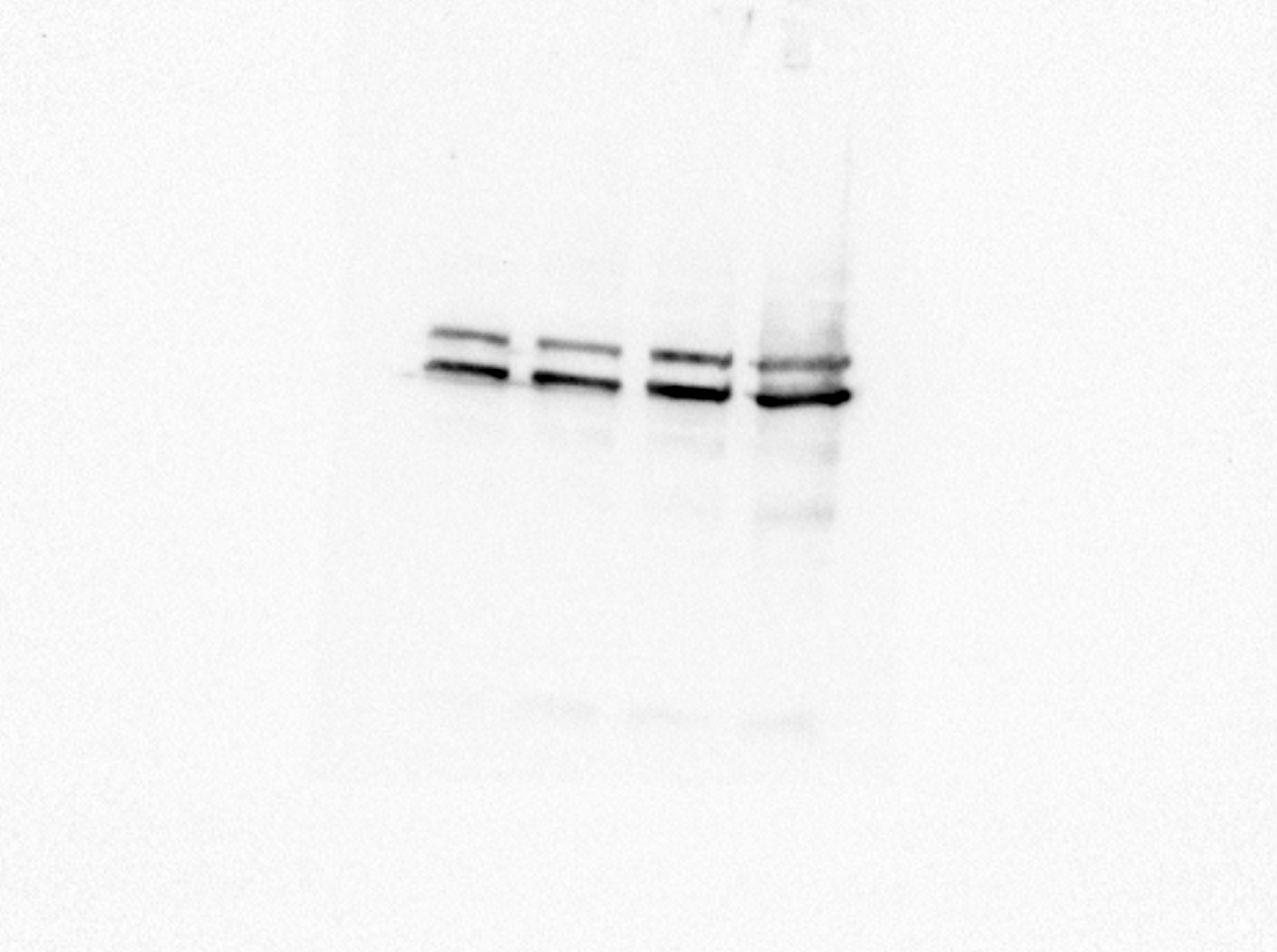

Supplement: Supplementary file 2 [file DataSheet2.zip › JSC-1/GAPDH.tif]

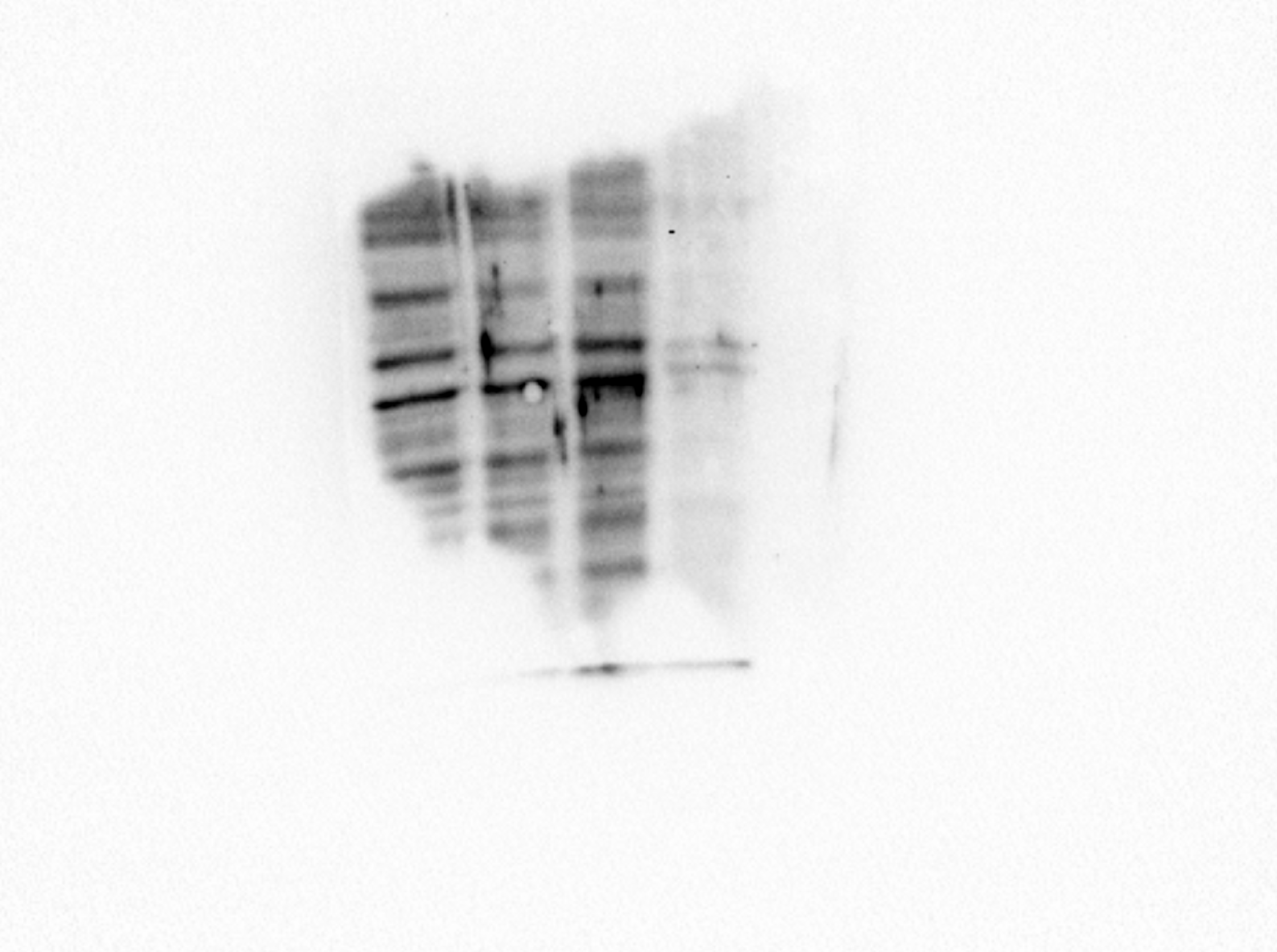

Supplement: Supplementary file 2 [file DataSheet2.zip › JSC-1/p_CHK-2.tif]

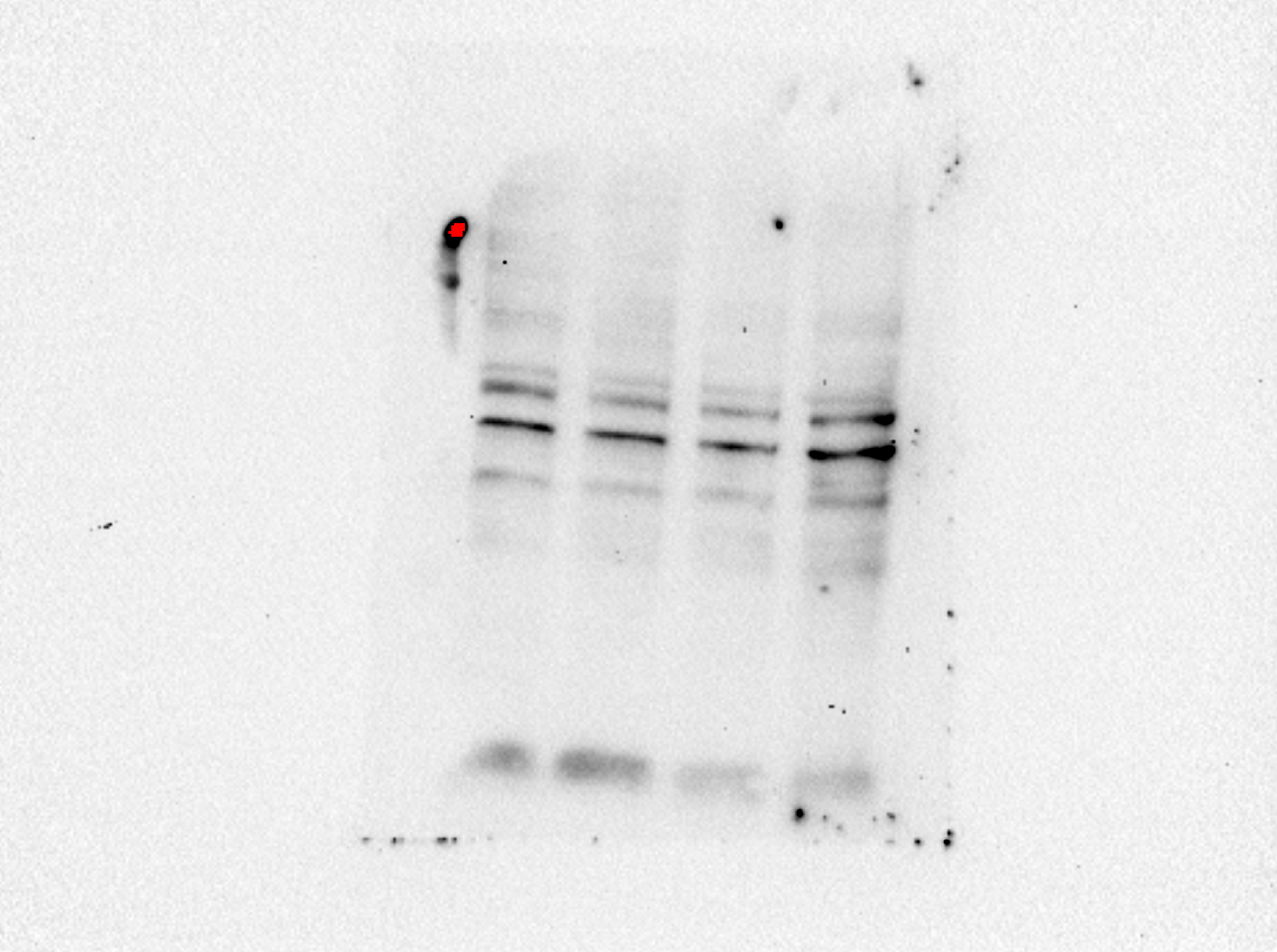

Supplement: Supplementary file 2 [file DataSheet2.zip › JSC-1/cMYC.tif]
